# SATB2‐LEMD2 interaction links nuclear shape plasticity to regulation of cognition‐related genes

**DOI:** 10.15252/embj.2019103701

**Published:** 2020-12-15

**Authors:** Patrick Feurle, Andreas Abentung, Isabella Cera, Nico Wahl, Cornelia Ablinger, Michael Bucher, Eduard Stefan, Simon Sprenger, David Teis, Andre Fischer, Aodán Laighneach, Laura Whitton, Derek W Morris, Galina Apostolova, Georg Dechant

**Affiliations:** ^1^ Institute for Neuroscience Medical University of Innsbruck Innsbruck Austria; ^2^ Institute of Biochemistry and Center for Molecular Biosciences University of Innsbruck Innsbruck Austria; ^3^ Institute for Cell Biology Medical University of Innsbruck Innsbruck Austria; ^4^ Department of Systems Medicine and Epigenetics German Center for Neurodegenerative Diseases (DZNE) Goettingen Germany; ^5^ Department of Psychiatry and Psychotherapy University Medical Center Goettingen Germany; ^6^ Neuroimaging, Cognition & Genomics (NICOG) Centre School of Psychology and Discipline of Biochemistry National University of Ireland Galway Galway Ireland

**Keywords:** chromatin, human cognitive ability, neuronal activity, nuclear envelope, SATB2, Chromatin, Epigenetics, Genomics & Functional Genomics, Membrane & Intracellular Transport, Neuroscience

## Abstract

*SATB2* is a schizophrenia risk gene and is genetically associated with human intelligence. How it affects cognition at molecular level is currently unknown. Here, we show that interactions between SATB2, a chromosomal scaffolding protein, and the inner nuclear membrane protein LEMD2 orchestrate the response of pyramidal neurons to neuronal activation. Exposure to novel environment *in vivo* causes changes in nuclear shape of CA1 hippocampal neurons via a SATB2‐dependent mechanism. The activity‐driven plasticity of the nuclear envelope requires not only SATB2, but also its protein interactor LEMD2 and the ESCRT‐III/VPS4 membrane‐remodeling complex. Furthermore, LEMD2 depletion in cortical neurons, similar to SATB2 ablation, affects neuronal activity‐dependent regulation of multiple rapid and delayed primary response genes. In human genetic data, LEMD2‐regulated genes are enriched for *de novo* mutations reported in intellectual disability and schizophrenia and are, like SATB2‐regulated genes, enriched for common variants associated with schizophrenia and cognitive function. Hence, interactions between SATB2 and the inner nuclear membrane protein LEMD2 influence gene expression programs in pyramidal neurons that are linked to cognitive ability and psychiatric disorder etiology.

## Graphical abstract

In cortical neurons, a chromosomal scaffold protein and an inner nuclear membrane protein orchestrate activity‐dependent nuclear shape changes and transcription of genes associated with cognitive function.

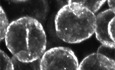

## Introduction

*SATB2* is a schizophrenia risk locus (Ripke *et al*, 2014; Li *et al*, 2017b) and is associated with human cognitive ability (Savage *et al*, 2018). We have recently shown that common variation in the genes regulated by SATB2 and in the genes encoding SATB2 protein interactors in the forebrain influence cognitive ability in the human general population and contribute to schizophrenia (Whitton *et al*, 2018; Cera *et al*, 2019). *SATB2* haploinsufficiency causes SATB2‐associated syndrome, an autosomal dominant genetic disorder characterized by severe developmental delay, intellectual disability, and craniofacial and dental abnormalities (Zarate *et al*, 2018). Studies in mouse models have revealed that SATB2 determines callosal projection neuron fate during cortex development (Alcamo *et al*, 2008; Britanova *et al*, 2008). In the adult mouse CNS, SATB2 is expressed in pyramidal neurons of all cortical layers and the CA1 field of the hippocampus (Zeisel *et al*, 2018) and is required for stabilization of Schaffer collateral long‐term potentiation and long‐term memory consolidation (Jaitner *et al*, 2016; Li *et al*, 2017a).

SATB2 binds to DNA via a homeodomain (HD), two CUT domains and a CUT‐like domain (FitzPatrick *et al*, 2003). An ubiquitin‐like domain (ULD) at the N‐terminus mediates its self‐association (Wang *et al*, 2012, Wang *et al* 2014). SATB2 homo‐dimeric or homo‐tetrameric complexes can modify the three‐dimensional genome folding by mediating the formation of chromatin loops (Wang *et al*, 2012). Accordingly, SATB2 has been referred to as a chromosomal scaffolding protein or genome organizer (Rajarajan *et al*, 2016). ChIP‐seq experiments have revealed an enrichment of SATB2 binding sites at transcription start sites (Jaitner *et al*, 2016), indicating a potent role as a transcriptional regulator. In fact, several studies have reported multiple dysregulated genes in *Satb2* knockout models (Alcamo *et al*, 2008; Zhou *et al*, 2012; Srivatsa *et al*, 2014; McKenna *et al*, 2015; Jaitner *et al*, 2016; Cera *et al*, 2019). In a recent proteomic study in mouse cortex (Cera *et al*, 2019), we identified interactions of SATB2 with nuclear lamina (NL)‐associated proteins, including lamins, inner nuclear membrane (INM) proteins of the LEM domain family, and barrier‐to‐autointegration factor (BAF) (Brachner & Foisner, 2011), indicating as yet undefined functions of SATB2 at the nuclear envelope.

Evidence from non‐neuronal cells suggests that the nuclear periphery contributes to spatial genome organization and gene expression (Czapiewski *et al*, 2016; Buchwalter *et al*, 2019). So far, it is unclear whether these mechanisms also operate in postmitotic neurons and whether they play a role in neuronal maturation and/or plasticity. The NL is a filamentous meshwork of A‐ and B‐type lamins attached to the nuclear envelope via interactions with INM proteins (Gruenbaum & Foisner, 2014). Mutations in genes encoding nuclear envelope proteins result in disorders called nuclear envelopathies that include various forms of muscular dystrophy, cardiomyopathy, lipodystrophy, neuropathy, and premature aging syndromes (Schreiber & Kennedy, 2013). Recently, a novel nuclear envelopathy has been discovered resulting from *de novo* mutation in LEM domain‐containing protein 2 (LEMD2) (Marbach *et al*, 2019). LEMD2 is an integral INM protein, considered as the most likely candidate for the chromatin‐binding component of the A tether (Thanisch *et al*, 2017). It has also been implicated in the structural organization of the nuclear envelope (Brachner *et al*, 2005) and in heterochromatin localization and silencing (Barrales *et al*, 2016). The reported LEMD2‐associated human phenotype displays several clinical similarities to the SATB2‐associated syndrome, in particular dental and craniofacial abnormalities, feeding difficulties, dysmorphic features, and low bone mineral density (Marbach *et al*, 2019). Given that LEMD2 co‐immunoprecipitates with SATB2 in cortical tissue lysates and the noticeable phenotypic overlap between the two syndromes, we used *Satb2* conditional mutants and *Lemd2* knockdown in cortical neurons to examine the physical and functional cooperation between these two proteins.

Here, we show that SATB2 is necessary for plastic changes in nuclear geometry occurring upon action potential (AP) bursting (Wittmann *et al*, 2009) and that both SATB2‐ and neuronal activity‐dependent nuclear envelope remodeling require LEMD2. We further demonstrate that LEMD2 depletion has a profound effect on gene expression in cortical neurons and that LEMD2‐ and SATB2‐regulated gene sets significantly overlap. LEMD2‐regulated genes are enriched for rare *de novo* mutations reported in schizophrenia and intellectual disability, as well as for common variants associated with schizophrenia and cognitive ability. Since genes functionally related to or targeted by SATB2 share similar enrichments (Whitton *et al*, 2018; Cera *et al*, 2019), our findings indicate that gene regulation by a SATB2‐LEMD2 chromatin tether in cortical neurons contributes to brain disorder etiology and human cognitive function.

## Results

### SATB2‐LEMD2 interaction requires the CUT‐like or CUT domains of SATB2 protein

**Figure 1:**
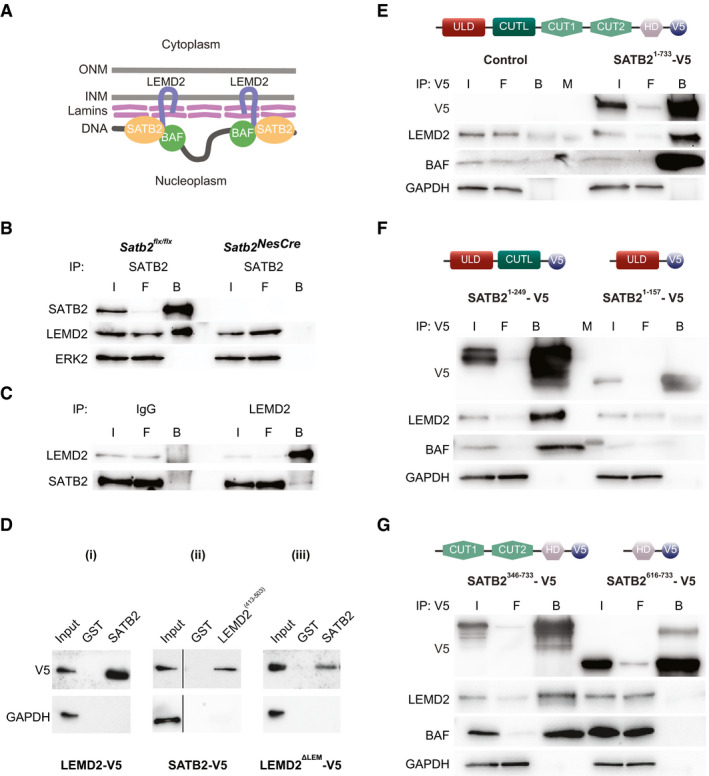
SATB2 interacts with the INM protein LEMD2 Model of a hypothetical NL‐chromatin tether containing SATB2. Schematic representation of the nuclear envelope consisting of two lipid bilayers, the inner nuclear membrane (INM) and outer nuclear membrane (ONM), and the lamin polymer underlying the INM (modified after Zuleger *et al*, 2011). Depicted are the INM protein LEMD2, lamins and BAF, identified as SATB2 interaction partners (Cera *et al*, 2019).Immunoprecipitation of SATB2 from mouse neonatal cortical lysates. LEMD2 was detected by Western blotting in the SATB2 immunoprecipitate from control but not SATB2‐deficient cortical lysate. The equal input of total protein from control and SATB2‐deficient cortical lysates was controlled by ERK2 detection. Representative images of the immunoblots are shown; I (Input), F (Flow‐through), B (Beads).Reverse immunoprecipitation with LEMD2 antibody and control IgG antibody, followed by WB detection of LEMD2 and SATB2. Lysates from primary cortical culture lysates were used.GST pull‐down assays. Representative images of *n* = 3 independent experiments are shown. GST pull‐down assay of transiently overexpressed V5‐tagged LEMD2 (i), SATB2 (ii), and LEMD2^ΔLEM^ (iii) from HeLa cell lysates using GST, GST‐SATB2, and GST‐LEMD2^(413–503)^ hybrid proteins.Immunoprecipitations using anti‐V5‐tag antibody from lysates of HeLa cells transfected with expression plasmids encoding V5‐tagged full‐length SATB2 (Satb2^1–733^) and EGFP as control. The equal input of total protein was controlled by GAPDH detection. Representative images of the immunoblots are shown; I (Input), F (Flow‐through), B (Beads), M (Molecular weight marker).The CUT‐like domain of SATB2 is required for the interaction with LEMD2. Immunoprecipitations using anti‐V5‐tag antibody from lysates of HeLa cells transfected with V5‐tagged Satb2^1–247^ and Satb2^1–157^ deletion mutants. LEMD2 and BAF were detected only in immunoprecipitates from HeLa cells transfected with the deletion mutant containing the CUT‐like domain (Satb2^1–247^). The equal input of total protein was controlled by GAPDH detection. Representative images of the immunoblots are shown; I (Input), F (Flow‐through), B (Beads), M (Molecular weight marker).CUT1 and CUT2 domains are required for the interaction with LEMD2. Immunoprecipitations using anti‐V5‐tag antibody from HeLa cells transfected with V5‐tagged Satb2^346–733^ and Satb2^616–733^ deletion mutants. LEMD2 and BAF were detected only in immunoprecipitates from HeLa cells expressing the CUT1 and CUT2 containing deletion mutant (Satb2^346–733^). Source data are available online for this figure.

Previously, we identified LEMD2 peptide sequences in SATB2 immunoprecipitates from mouse cortical lysates by mass spectrometry (Cera *et al*, 2019). To further explore SATB2‐LEMD2 interaction (Fig 1A), we first validated these mass spectrometry data by independent co‐immunoprecipitation of SATB2 from mouse cortical lysates followed by Western blotting. We identified LEMD2 in SATB2 immunoprecipitates from *Satb2^flx^* ^/^ *^flx^* (floxed) but not from *Satb2^flx^* ^/^ *^flx^::Nes‐Cre* knockout (*Satb2^NesCre^*) cortical lysates (Appendix Fig S1, Fig 1B). In a reverse co‐immunoprecipitation, SATB2 was pulled‐down from cortical culture lysates by a LEMD2 antibody, but not by a control rabbit IgG (Fig 1C). Next, we confirmed SATB2‐LEMD2 interaction by GST pull‐down assays in HeLa cells, which are devoid of endogenous SATB2 (Appendix Fig S2 and Fig 1D). Recombinant V5‐tagged LEMD2 was precipitated from HeLa cell lysates with a recombinant, bacterially expressed GST‐SATB2 fusion protein but not with GST alone (Fig 1D, i). Likewise, V5‐tagged SATB2 from HeLa cell lysates was co‐precipitated with GST‐LEMD2^413–503^ hybrid protein but not with GST alone (Fig 1D, ii). LEM domain proteins are defined by a common 40 AA helix‐loop‐helix motif (LEM), which mediates binding to the DNA cross‐bridging protein BAF (Brachner & Foisner, 2011). Since we previously identified also BAF as a SATB2 interactor (Cera *et al*, 2019), we tested whether the interaction between SATB2 and LEMD2 depends on the presence of BAF. We employed a V5‐tagged LEMD2 deletion mutant that lacks the LEM domain (LEMD2^ΔLEM^) and thus is incapable of interacting with BAF (Shumaker *et al*, 2001). BAF binding is not necessary for LEMD2‐SATB2 interaction since LEMD2^ΔLEM^, like the full‐length LEMD2, was co‐precipitated with a GST‐SATB2 fusion protein but not with GST alone (Fig 1D, iii).

To define protein domains of SATB2, which are necessary for the interaction with LEMD2, we generated several SATB2 deletion constructs: (i) SATB2^1–157^, containing only the ULD domain; (ii) SATB2^1–249^, lacking the two CUT domains and the HD; (iii) SATB2^346–733^, lacking the ULD and the CUT‐like domain; and (iv) SATB2^616–733^, containing only the HD domain. HeLa cells were transfected with plasmids encoding V5‐tagged full‐length SATB2^1–733^ or the corresponding V5‐tagged SATB2 deletion mutants. pEGFP‐C1 plasmid served as a negative control. LEMD2 and BAF were detected in V5 immunoprecipitates from HeLa cells transfected with the full‐length SATB2^1–733^ but not with the control plasmid (Fig 1E), thus confirming the interaction observed in neonatal cortical lysates. SATB2‐LEMD2 and SATB2‐BAF interactions were also detected in HeLa cells transfected with the SATB2 deletion mutants that contain either the two CUT domains (SATB2^346–733^) or the CUT‐like domain (SATB2^1–249^) (Fig 1F and G). In contrast, interactions of SATB2 with LEMD2 or BAF were not observed in lysates of HeLa cells expressing SATB2 deletion mutants that lack the CUT‐like as well as the two CUT domains and contain only the ULD domain (SATB2^1–157^) or the HD domain (SATB2^616–733^) (Fig 1F and G). Taken together, our data demonstrate that LEMD2 is a binding partner of SATB2 in pyramidal neurons and that this interaction requires the CUT‐like, CUT1, and/or CUT2 domains of the SATB2 protein.

### SATB2 is required for synaptic activity‐triggered nuclear membrane remodeling of pyramidal neurons

**Figure 2:**
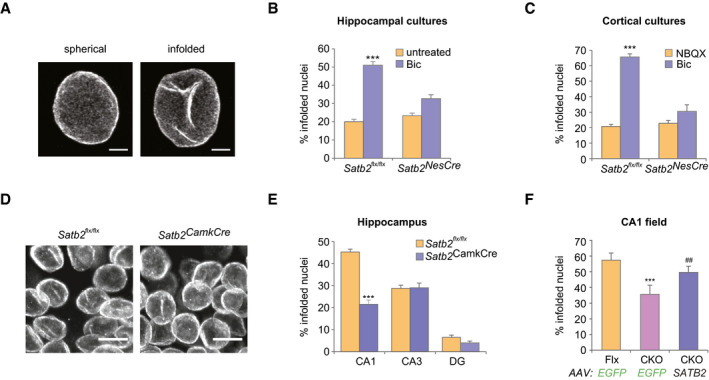
SATB2 determines nuclear envelope structural plasticity in neurons Representative images of *z*‐axis projected stacks of confocal images of hippocampal neuronal nuclei immunostained for Lamin B2. Examples of spherical and infolded nuclei are shown. Scale bars: 3 μm.Activity‐induced formation of nuclear infoldings is abolished in SATB2‐deficient hippocampal neurons. Bic‐induced AP bursting for 1 h caused a significant increase in the percentage of infolded nuclei in DIV10 hippocampal cultures derived from Satb2^flx/flx^ mice but not from Satb2^flx/flx^::Nes‐Cre mice (*Satb2^NesCre^*) (*n* = 6 independent primary cultures, two‐way ANOVA, *F*_1,20_ = 9.51, significant interaction *P* = 0.0058, simple main effects analysis, Satb2^flx/flx^ cultures, Bic‐treated vs untreated *P* = 0.0000043, *Satb2^NesCre^* cultures, Bic‐treated vs untreated *P* > 0.05, adjustment for multiple comparisons: Bonferroni, number of analyzed nuclei: Satb2^flx/flx^ cultures, untreated—680, Satb2^flx/flx^ cultures, Bic‐treated—711, *Satb2^NesCre^*, untreated—755, *Satb2^NesCre^*, Bic‐treated—720). Data are presented as mean ± SEM, ***P < 0.001.Activity‐induced formation of nuclear infoldings is impaired in SATB2‐deficient cortical neurons. DIV14 cortical cultures form Satb2^flx/flx^ and *Satb2^NesCre^* mice were silenced with NBQX for 1 h followed by stimulation with Bic for 1 h. The percentage of infolded nuclei following AP bursting was increased in control Satb2^flx/flx^ cultures but not in SATB2‐deficient cultures (*n* = 3–4 independent primary cultures, two‐way ANOVA, *F*_1,10_ = 39.124, significant interaction *P* = 0.000094, simple main effects analysis, Satb2^flx/flx^ cultures: Bic‐treated vs NBQX‐treated *P* = 7.66E‐07, *Satb2^NesCre^* cultures: Bic‐treated vs NBQX‐treated *P* > 0.05, adjustment for multiple comparisons: Bonferroni, number of analyzed nuclei: Satb2^flx/flx^ cultures, NBQX—606, Satb2^flx/flx^ cultures, Bic—910; *Satb2^NesCre^* cultures, NBQX—604, *Satb2^NesCre^* cultures, Bic—835). Data are presented as mean ± SEM, ***P < 0.001.Confocal images (*z*‐axis‐projected stacks) of Lamin B2 stained neuronal nuclei from the CA1 pyramidal cell layer of adult *Satb2^flx^* ^/^ *^flx^* and *Satb2^flx^* ^/^ *^flx^::CamK2a‐Cre* (*Satb2^CamkCre^*) mice. Scale bars: 10 μm.Analysis of the percentage of infolded nuclei in the hippocampus of Satb2^flx/flx^ mice vs *Satb2^CamkCre^* mice. In Satb2^flx/flx^ mice, the number of infolded nuclei was significantly higher in the CA1 area compared to the CA3 area (*P* = 0.000001) and DG (*P* = 1.8 E‐12). In *Satb2^CamkCre^* mice, the number of infolded nuclei in the CA1 area was significantly reduced compared to the corresponding number in littermate controls (*P* = 1.9 E‐09). No significant differences were observed in this number in the CA3 and DG between *Satb2^CamkCre^* mice and floxed controls (*n* = 4 mice, two‐way ANOVA, significant interaction, *F*_2,18_ = 47.81, *P* = 0.00001, simple main effects analysis, CA1 area, *Satb2^flx^* ^/^ *^flx^* vs CKO mice *P* = 1.9 E‐09, CA3 area, *Satb2^flx^* ^/^ *^flx^* vs CKO mice *P* = 0.909, DG, *Satb2^flx^* ^/^ *^flx^* vs CKO mice *P* = 0.262, adjustment for multiple comparisons: Bonferroni). Number of analyzed nuclei: *Satb2^flx^* ^/^ *^flx^* mice: 469 (CA1), 225 (CA3), 665 (DG); *Satb2^CamkCre^*: 508 (CA1), 224 (CA3), 575 (DG). Data are presented as mean ± SEM, *** *P* < 0.001.Analysis of the percentage of infolded nuclei in the CA1 pyramidal cell layer of *Satb2^CamkCre^* mice vs littermate controls after stereotaxic injections of AAV‐SATB2‐*V5* or AAV8‐EGFP. Viral delivery of SATB2 resulted in restoration of the number of infolded nuclei in the CA1 pyramidal cell layer of *Satb2^CamkCre^* mice (*n* = 3–5 mice, one‐way ANOVA followed by Hochberg *post hoc* test, *F*_2,9_ = 33.1, *Satb2^CamkCre^*::AAV‐SATB2 vs *Satb2^flx^* ^/^ *^flx^*::AAV‐EGFP, *P* = 0.0012; *Satb2^flx^* ^/^ *^flx^*::AAV‐EGFP vs *Satb2^CamkCre^*::AAV‐EGFP, *P* = 0.00008, *Satb2^flx^* ^/^ *^flx^*::AAV‐EGFP vs *Satb2^CamkCre^*::AAV‐SATB2, *P* = 0.072). Number of analyzed nuclei: 388 (*Satb2^flx^* ^/^ *^flx^*::AAV‐EGFP), 560 (*Satb2^CamkCre^*::AAV‐EGFP), 546 (*Satb2^CamkCre^*::AAV‐SATB2). Data are presented as mean ± SEM, ***P < 0.001 compared to *Satb2^flx^* ^/^ *^flx^*::AAV‐EGFP, ^##^ *P* < 0.01, compared to *Satb2^CamkCre^*::AAV‐EGFP. Source data are available online for this figure.

LEMD2 has an established role in the maintenance of normal nuclear morphology (Ulbert *et al*, 2006). It interacts with other regulators of nuclear shape such as Lamin A/C (Brachner *et al*, 2005) and emerin (Huber *et al*, 2009), and mutations in *Lemd2* cause a misshaped nucleus with invaginations and lobulations (Gerull *et al*, 2018; Marbach *et al*, 2019). In non‐neuronal cells, nuclear shape is affected by interactions between INM proteins and chromatin (Polychronidou & Grobhans, 2011; Czapiewski *et al*, 2016). Therefore, we asked whether SATB2, as a chromatin scaffolding protein and a newly identified LEMD2‐binding partner in neurons, controls neuronal nuclear shape. In pyramidal neurons, dramatic transformations in the geometry of the cell nucleus, visualized as deep infoldings/invaginations of the nuclear membrane, occur upon AP bursting (Wittmann *et al*, 2009; Queisser *et al*, 2011) (Fig 2A). Thus, we tested whether SATB2 affects this synaptic activity‐triggered formation of nuclear infoldings. We compared the number of infolded nuclei in hippocampal cultures derived from *Satb2* floxed or *Satb2^NesCre^* mice under resting conditions and after stimulation with bicuculline (Bic), a GABA‐A receptor antagonist that causes bursts of AP firing (Hardingham *et al*, 2002). Lamin B2 immunostaining showed that in *Satb2* floxed cultures, neuronal activity caused a robust increase in the percentage of neurons with infolded nuclei, as previously reported in wild‐type cultures by Wittmann *et al* (2009). In contrast, in *Satb2^NesCre^* cultures, AP bursting had no effect on nuclear geometry (Fig 2B). As phospho‐MSK1 immunoreactivity has previously been shown to correlate with the quantity of nuclear infoldings (Wittmann *et al*, 2009), we also examined phospho‐MSK1 levels in *Satb2^flx^* ^/^ *^flx^* vs *Satb2^NesCre^* cultures. We did not detect any difference in phospho‐MSK1 immunoreactivity between floxed and knockout cultures (Appendix Fig S3), indicating that SATB2 deficiency disrupts hippocampal neuron ability to respond to synaptic activity by changes in nuclear shape independently of MSK1/2 activation. Furthermore, similar to hippocampal neurons, *Satb2* ablation in primary cortical neurons also affected nuclear shape, since Bic treatment significantly increased the percentage of infolded nuclei in floxed but not in *Satb2^NesCre^* cortical neurons (Fig 2C).

As cortex and hippocampus contain infolded neuronal nuclei *in vivo* (Buschmann & LaVelle, 1981; Wittmann *et al*, 2009), we next explored whether SATB2 plays a role in the formation of nuclear infoldings *in vivo.* Lamin B2 immunostaining was used to quantitatively analyze the nuclear geometry in adult hippocampal and cortical tissue. In the hippocampus of floxed animals, we found the highest number of infolded nuclei in CA1 (45.7 ± 1.8%, Fig 2E), where SATB2 is expressed (Zeisel *et al*, 2018). Lower numbers of infolded nuclei were detected in CA3 (27.3 ± 0.3%) and dentate gyrus (DG, 7.3 ± 0.9%) which are devoid of SATB2. We then compared the number of infolded nuclei in the hippocampus and cortex of adult *Satb2^flx^* ^/^ *^flx^::Camk2a‐Cre* (*Satb2^CamkCre^*) mutants vs *Satb2^flx^* ^/^ *^flx^* controls (Jaitner *et al*, 2016). We found significantly fewer nuclei with infoldings in the CA1 field of knockout vs floxed mice (Fig 2D and E). By contrast, the number of nuclei with infoldings in the CA3 field and DG, where *Satb2* is not expressed, was not different between *Satb2^CamkCre^* and control mice. Fewer infolded nuclei were also identified in layer 2/3 of the visual cortex of SATB2‐deficient vs control floxed mice (unpaired *t*‐test, *t*(5) = 4.158, *P* = 0.0088, *n* = 4 floxed mice, *n* = 3 *Satb2^CamkCre^* mice, number of analyzed nuclei: 818 in floxed mice, 729 in *Satb2^CamkCre^* mice).

**Figure EV1:**
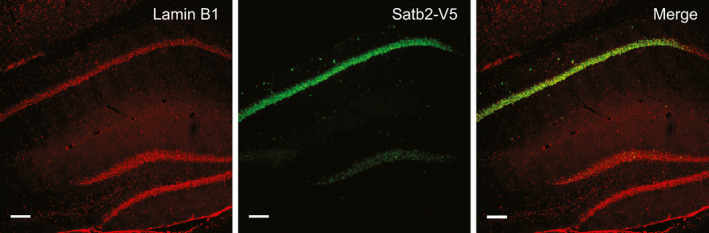
Re‐introduction of V5‐tagged SATB2 into the adult dorsal hippocampus of Satb2 conditional mutants rAAV8‐hSyn‐*Satb2‐V5* virus was injected into the dorsal hippocampus of *Satb2^CamKCre^* knockout mice. Lamin B2 staining (left panel) was used to mark cell nuclei in hippocampus. V5 immunoreactivity was used to detect expression of SATB2‐V5 in hippocampus (middle panel). Overlay of both pictures (right panel) demonstrates re‐expression of SATB2 in CA1‐neuronal nuclei in SATB2‐deficient mice. Representative images are shown. Scale bars: 100 μm.

To test whether restoring SATB2 expression can rescue infolding formation in CA1 of adult *Satb2^CamkCre^* mice *in vivo*, we employed AAV‐mediated gene delivery. Viral vectors encoding SATB2 or EGFP were injected into the dorsal hippocampus of *Satb2^CamkCre^* and *Satb2^flx^* ^/^ *^flx^* mice (Fig EV1). Injection with control AAV8‐EGFP did not rescue the loss of infoldings in CA1 pyramidal neurons of *Satb2^CamkCre^* mice (Fig 2F). By contrast, the percentage of infolded nuclei in the CA1 area of SATB2‐deficient mice transduced with AAV8‐SATB2 was restored to levels that were not statistically different from the levels in AAV8‐EGFP‐injected floxed mice, demonstrating a complete rescue of the formation of nuclear infoldings by re‐introduction of SATB2 into the CA1 of adult Satb2 knockout mice (Fig 2F). Taken together, our results support a requirement for SATB2 in the induction of nuclear infoldings upon AP bursting *in vitro* and establish SATB2 as a regulator of neuronal nuclear geometry *in vivo*.

### SATB2 cooperates with LEMD2 to regulate nuclear plasticity

**Figure 3:**
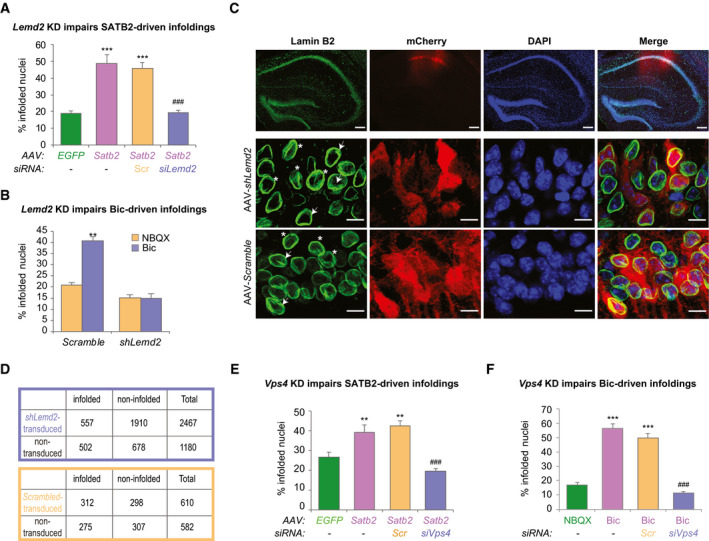
SATB2 cooperates with LEMD2 and the AAA‐ATPase VPS4 to regulate nuclear infolding formation Nuclear infolding triggered by SATB2 overexpression requires LEMD2. Analysis of the percentage of infolded nuclei in DIV10 hippocampal neurons upon *Satb2* overexpression and *Lemd2* knockdown. AAV‐SATB2*‐*transduced neurons (both non‐transfected and scrambled siRNA‐transfected, Scr) exhibited higher percentage of infolded nuclei compared to AAV‐EGFP*‐*transduced neurons. In contrast, LEMD2‐depleted AAV‐SATB2*‐*transduced cultures showed similar percentage of infolded nuclei to AAV‐EGFP*‐*transduced neurons (*n* = 3–4 independent primary cultures; one‐way ANOVA *F*_3,11_ = 30.982, *P* = 0.000011; Hochberg *post hoc* test, AAV‐EGFP vs AAV‐SATB2 *P *=* *0.0001, AAV‐EGFP vs AAV‐SATB2 + S*cr* *P* = 0.0001, AAV‐EGFP vs AAV‐SATB2 + *siLemd2* *P* = 0.9999, AAV‐SATB2 + *Scr* vs AAV‐SATB2 + *siLemd2* *P* = 0.0001). Number of nuclei analyzed: 460 (AAV‐EGFP), 466 (AAV‐SATB2), 537 (AAV‐SATB2 + *Scr)*, 597 (AAV‐SATB2* *+* *siLem2). Data are presented as mean ± SEM, ***P < 0.001 compared to AAV‐EGFP; ^###^ *P* < 0.001 compared to AAV‐SATB2 + *Scr*.Activity‐induced formation of nuclear infoldings is impaired in LEMD2‐depleted cortical neurons. Bic‐triggered AP bursting for 1 h caused a significant increase in the percentage of infolded nuclei in cortical neurons transduced with AAV‐scrambled but not with AAV‐*shLemd2* virus (*n* = 5 independent primary cultures, two‐way ANOVA, *F*_1,16_ = 8.35, significant interaction *P* = 0.0107, simple main effects analysis: AAV‐scrambled‐transduced cultures, Bic‐treated vs untreated *P* = 0.0049, AAV‐*shLemd2‐*transduced cultures, Bic‐treated vs untreated *P *> 0.99, adjustment for multiple comparisons: Bonferroni). Data are presented as mean ± SEM, **P < 0.01 compared to NBQX‐treated cultures.LEMD2 is required for the nuclear envelope infoldings of CA1 pyramidal neurons *in vivo*. Top panel: representative confocal images (*z*‐axis‐projected stack) of Lamin B2/DAPI‐stained coronal brain sections from wild‐type mice after stereotaxic injection of AAV‐shRNA viruses (AAV‐*shLemd2*‐mCherry or AAV‐scrambled‐mCherry) into the dorsal hippocampus. Merged, colocalization of the three signals. Scale bars: 50 μm. Middle panel: higher magnification images of AAV‐*shLemd2‐*transduced CA1 pyramidal neurons. Arrows show examples of mCherry‐positive nuclei that are devoid of nuclear infoldings, whereas stars denote non‐transduced infolded nuclei. Lower panel: higher magnification images of AAV‐scrambled *siRNA*‐transduced CA1 pyramidal neurons. Arrows show examples of infolded, mCherry‐positive nuclei; stars denote infolded, non‐transduced neurons. Scale bars (middle and lower panels): 10 μm.2 × 2 contingency tables showing the number of analyzed CA1 pyramidal neuron nuclei grouped by category: (top) AAV‐*shLemd2* transduced/non‐transduced (*n* = 3 mice), (bottom) AAV‐*scrambled shRNA‐*transduced/non‐transduced (*n* = 2 mice). AAV‐*Lemd2* s*hRNA*‐transduced nuclei were less likely to be infolded than the non‐transduced nuclei (Fisher's exact test, *P* < 1.00E‐15, OR = 0.39, 95% confidence interval: 0.34–0.45). The percentage of infolded AAV‐*scrambled shRNA‐*transduced nuclei was not significantly different from the percentage of infolded non‐transduced nuclei in the same optical field (Fisher's exact test, *P* = 0.1830).Analysis of the percentage of infolded nuclei in DIV10 hippocampal neurons upon SATB2 overexpression and *Vps4a*/*Vps4b* knockdown. AAV‐SATB2*‐*transduced neurons (non‐transfected or scrambled siRNA (Scr)‐transfected) exhibited higher percentage of infolded nuclei compared to AAV‐EGFP*‐*transduced neurons. In contrast, VPS4‐depleted AAV‐SATB2*‐*transduced cultures showed similar number of infolded nuclei to AAV‐EGFP*‐*transduced neurons (*n* = 4 independent primary cultures, one‐way ANOVA followed by Tukey *post hoc* test, *F*_3,11_ = 15.866, *P* = 0.00026, AAV‐EGFP vs AAV‐SATB2 *P *=* *0.0104, AAV‐*EGFP* vs AAV‐SATB2 + *Scr P *=* *0.0028, AAV‐EGFP vs AAV‐SATB2 + *siVps4 P* = 0.605, AAV‐SATB2 + *Scr* vs AAV‐SATB2 + *siVps4 P* = 0.0007). Number of nuclei analyzed: 594 (AAV‐EGFP), 657 (AAV‐SATB2), 612 (AAV‐SATB2 + *Scr*), 428 (AAV‐SATB2 + *siVps4*). Data are presented as mean ± SEM, **P < 0.01 compared with AAV‐*EGFP*; ^###^ *P* < 0.001 compared with AAV‐SATB2 + *Scr*.Activity‐induced formation of nuclear infoldings is impaired in VPS4‐depleted cortical neurons. Upon AP bursting, the percentage of infolded nuclei was increased in non‐transfected cultures and in cultures transfected with scramble siRNA (*Scr*) but not with *siVps4* (*n* = 3–4 independent primary cultures, one‐way ANOVA followed by Tukey *post hoc* test, *F*_3,8_ = 90.525, *P* = 1.63E‐06, Bic‐treated vs NBQX‐treated *P* = 0.000012, *Scr* Bic‐treated vs NBQX‐treated *P* = 0.000052, *siVps4* Bic‐treated vs NBQX‐treated *P* = 0.396, *siVps4* Bic‐treated vs *Scr* Bic‐treated *P* = 0.000015). Number of analyzed nuclei: 474 (NBQX), 456 (Bic), 409 (Bic + *Scr*), 383 (Bic + *siVps4*). Data are presented as mean ± SEM, ***P < 0.001 compared to NBQX‐treated, ^###^ *P* < 0.001 compared to *Scr* Bic‐treated. Source data are available online for this figure.

**Figure EV2:**
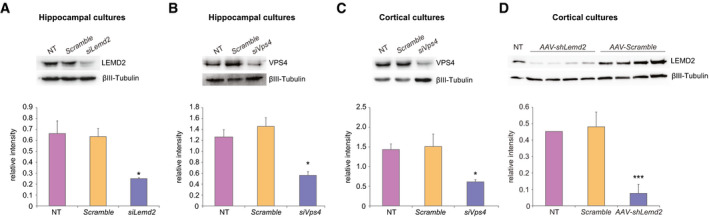
Gene silencing of *Lemd2*, *Vps4a* and *Vps4b* in primary neuronal cultures Gene silencing of *Lemd2* in hippocampal cultures by siRNA. Upper panel: Representative Western blot for LEMD2 protein in primary hippocampal neurons, *NT* (non‐transfected), *Scramble* (transfected with control siRNA), *siLemd2* (transfected with siRNA against *Lemd2*), Lower panel: Western blot quantification of LEMD2 in hippocampal cultures, *n* = 4 independent primary cultures. One‐way Welch’s ANOVA followed by Tukey *post hoc* test, *F*_2,9_ = 16.783, *P* = 0.011, NT vs *siLemd2* *P* = 0.013, *Scramble* vs *siLemd2* *P* = 0.019. Data are presented as mean ± SEM, *P < 0.05 compared to NT.Gene silencing of *Vps4* in hippocampal cultures by siRNA. Upper panel: Representative Western blot for VPS4 protein level in primary hippocampal neurons. *NT* (non‐transfected), *Scramble* (transfected with control siRNA), *siVps4* (transfected with siRNAs against *Vps4a* and *Vps4b)*. Lower panel: Western blot quantification of VPS4 protein level in primary hippocampal cultures, *n* = 3 independent experiments, one‐way Welch’s ANOVA followed by Tukey *post hoc* test, *F*_2,6_ = 14.226, *P* = 0.027, NT vs *siVps4* *P* = 0.063, *Scramble* vs *siLemd2* *P* = 0.045. Data are presented as mean ± SEM, *P < 0.05 compared to *scramble*.Gene silencing of *Vps4* in cortical cultures by siRNA. Upper panel: Representative Western blot for VPS4 protein level in primary cortical neurons. *NT* (non‐transfected), *scramble* (transfected with control siRNA), *Vps4* (transfected with siRNA against *Vps4a* and *Vps4b*), Lower panel: Western blot quantification of VPS4 protein level in primary cortical cultures, *n* = 3 independent experiments, one‐way ANOVA followed by Tukey *post hoc* test, *F*_2,8_ = 9.423, *P* = 0.014, NT vs *siVps4* *P* = 0.041, *scramble* vs *siVps4* *P* = 0.015. Data are presented as mean ± SEM, *P < 0.05 compared to NT.Gene silencing of *Lemd2* in cortical cultures by shRNA. Upper panel: Western blot for LEMD2 in primary cortical neurons, *NT* (non‐transfected), *Scramble* (transfected with control shRNA), *AAV‐shLemd2* (transfected with shRNA against *Lemd2*). Lower panel: Western blot quantification of LEMD2 protein level, *n* = 4 independent experiments, unpaired *t*‐test*, t*(6) = 5.98*, P* = 0.000979. Data are presented as mean ± SEM, ***P < 0.001 compared to *Scramble*. Source data are available online for this figure.

Compared to cortical cultures, SATB2 expression levels are lower in hippocampal primary cultures. This allowed us to ask whether SATB2 overexpression by AAV‐mediated gene delivery in hippocampal neurons is sufficient to cause nuclear envelope infoldings under resting conditions. Transduction with AAV8‐SATB2 caused a significant increase in the number of infolded nuclei compared to transduction with AAV8‐EGFP (Fig 3A). We then explored whether SATB2‐dependent nuclear remodeling requires LEMD2. Early embryonic lethality of the available *Lemd2* knockout mice (Tapia *et al*, 2015) precludes analyses of LEMD2‐deficient postnatal neurons. We therefore knocked down *Lemd2* expression in primary neurons by siRNA‐ or shRNA‐mediated gene silencing (Grünewald *et al*, 2019) (Fig EV2A and D). In control neurons transfected with scrambled siRNA, SATB2 viral delivery caused a robust increase in the number of infolded nuclei, whereas in LEMD2‐depleted neurons SATB2 overexpression had no effect on nuclear infoldings (Fig 3A). Moreover, in primary cortical neurons, AAV‐shRNA‐mediated *Lemd2* knockdown, similar to *Satb2* knockout (Fig 2B), completely blocked Bic‐induced formation of nuclear infoldings, whereas transduction with control virus encoding a scrambled shRNA did not affect synaptic activity‐triggered increase in the number of infolded nuclei (Fig 3B). We conclude that LEMD2 is critical for both activity‐ and SATB2‐induced nuclear envelope plasticity in cortical and hippocampal neurons *in vitro*.

Next, we assessed the impact of shRNA‐mediated *Lemd2* knockdown on the nuclear morphology of CA1 pyramidal neurons *in vivo*. AAV viruses (encoding either *Lemd2* or scrambled shRNA) were injected into the dorsal hippocampus of 3‐month‐old wild‐type mice, and 4 weeks later, the shape of CA1 neuronal nuclei was analyzed by Lamin B2 immunostaining (Fig. 3C). AAV‐*Lemd2* shRNA‐transduced nuclei, detected by the presence mCherry co‐expressed from the virus, were less likely to be infolded than non‐transduced mCherry‐negative nuclei observed in the same optical field (Fig 3C and D). The percentage of infolded nuclei in neurons transduced with scrambled shRNA was not statistically different from non‐transduced nuclei in the same optical field (Fig 3D). Together, these data demonstrate a critical role of LEMD2 in shaping pyramidal neuron nuclei both *in vitro* and *in vivo.*

### SATB2‐dependent changes in nuclear morphology depend on ESCRT‐III and VPS4 ATPase activity

**Figure EV3:**
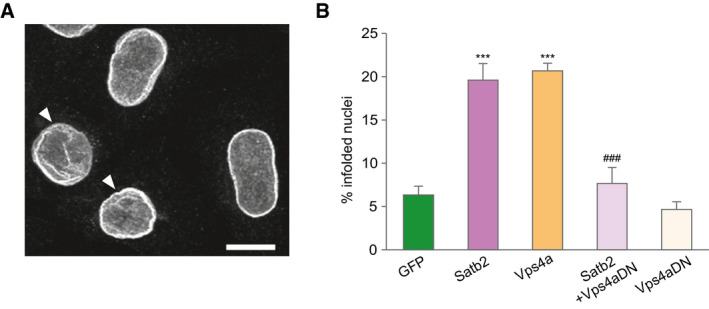
5 ESCRT‐III/VPS4 complex is required for SATB2‐triggered nuclear infolding formation in HeLa cells Confocal image (*z*‐axis projection of confocal image stack) of HeLa cell nuclei immunostained for Lamin B2. Arrowheads indicate infolded nuclei. Scale bar: 10 μm.Percentage of infolded nuclei in HeLa cells after ectopic expression of *Satb2*, *Vps4a‐GFP*, a dominant‐negative *Vps4a* mutant fused to GFP (*Vps4aDN‐GFP*), and a combination of *Satb2* and *Vps4aDN‐GFP*. The number of infolded nuclei is significantly increased in both *Satb*2‐ and *Vps4a*‐transfected cells compared with GFP‐transfected cells. Expression of the dominant‐negative *Vps4a* mutant abolished the increase in the number of infolded nuclei induced by SATB2, *n* = 3–5 independent experiments, ANOVA followed by Tukey *post hoc* test, *F*_4,14_ = 25.4, *GFP* vs *Satb2*, *P* < 0.0001, *GFP* vs *Vps4a*, *P* < 0.0001, *Satb2* vs *Satb2 *+* Vps4aDN*, *P* = 0.0006. Number of analyzed nuclei: 521 (*GFP*), 586 (*Satb2*), 302 (*Vps4a*), 345 (*Satb2*/*Vps4aDN*), 317 (*Vps4aDN*). Data are presented as mean ± SEM, ***P < 0.0001 compared to *GFP*; ^###^ *P* < 0.001 compared to *Satb2*.

LEMD2 is known to recruit components of the ESCRT‐III membrane‐remodeling machinery (Christ *et al*, 2017) to mediate nuclear envelope resealing (Olmos *et al*, 2015; Vietri *et al*, 2015; Webster *et al*, 2016; Gu *et al*, 2017) and to control heterochromatin‐nuclear envelope attachments (Pieper *et al*, 2020). In our previous proteomic study, we identified subunits of the ESCRT‐III complex (CHMP4B and CHMP3) and the AAA‐ATPase VPS4 as SATB2 interaction partners in cortical neurons (Cera *et al*, 2019). Hence, we set out to examine whether ESCRT‐III and VPS4 are required for the formation of SATB2‐induced nuclear invaginations. To suppress ESCRT‐III‐dependent membrane remodeling, we used siRNA‐mediated knockdown of the AAA‐ATPases VPS4A and VPS4B (Fig EV2B and C). As expected, in neurons transfected with scrambled siRNA, SATB2 overexpression increased the number of infolded nuclei compared to EGFP‐transduced cultures (Fig 3E). In *siVps4a*/*Vps4b*‐transfected cells, the percentage of infolded nuclei remained unchanged upon SATB2 viral transduction (Fig 3E). VPS4 depletion also abolished neuronal activity‐induced nuclear infolding formation (Fig 3F). In HeLa cells, the ectopic expression of either SATB2 or VPS4 significantly increased the number of infolded nuclei compared with mock‐transfected cultures (Fig EV3A and B). Co‐expression of SATB2 together with VPS4A^E288Q^‐GFP, a dominant‐negative VPS4A mutant that binds to ATP but fails to hydrolyze it (Babst *et al*, 1997), abolished the increase in the number of infolded nuclei caused by SATB2 overexpression (Fig EV3B). These results implicate the AAA‐ATPase VPS4 in nuclear envelope remodeling and suggest a novel role of ESCRT‐III and VPS4 at the nuclear periphery in postmitotic cells.

### LEMD2 and SATB2 regulate overlapping gene sets in primary cortical neurons

**Figure EV4:**
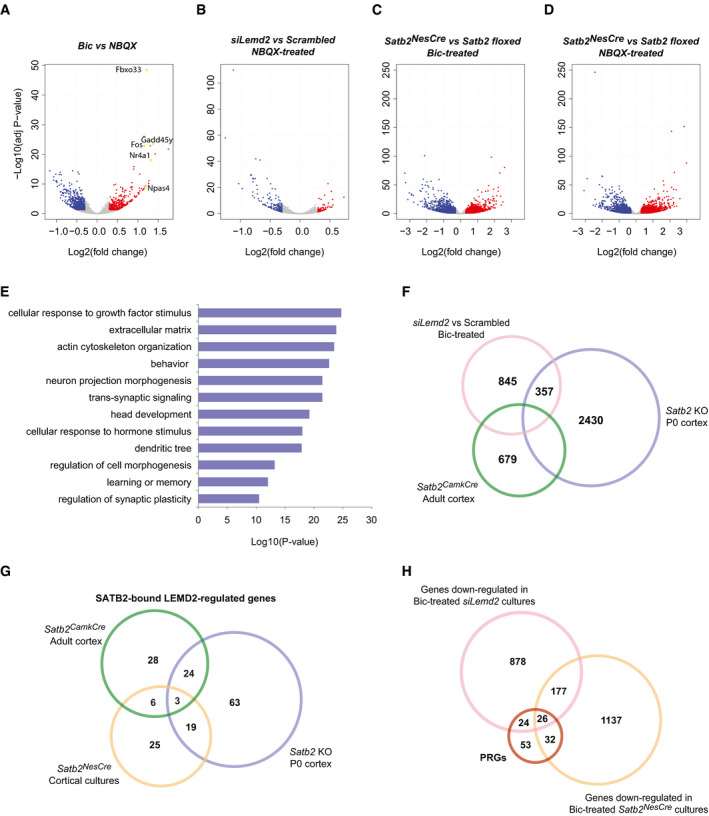
LEMD2 and SATB2 coregulate neuronal gene transcription A“Volcano plot” of statistical significance against fold change (FC) between silenced (NBQX‐treated) and active (Bic‐treated) primary cortical cultures from *Satb2* floxed mice. The differentially expressed genes (adjusted *P*‐value < 0.05, 0.3 FC cut‐off) are indicated in red (up‐regulated) and in blue (down‐regulated), *n* = 5–7 independent primary cultures. Examples of some IEGs strongly up‐regulated upon Bic treatment are marked and highlighted in yellow.B–D“Volcano plots” illustrating the differential gene expression between NBQX‐treated *siLemd2‐* vs scrambled siRNA‐transfected cortical cultures (B), Bic‐treated *Satb2^NesCre^* vs Satb2 floxed cultures (C), and NBQX‐treated *Satb2^NesCre^* vs Satb2 floxed cultures (D). The differentially expressed genes (adjusted *P*‐value < 0.05, 0.3 FC cut‐off) are indicated in red (up‐regulated) and in blue (down‐regulated), *n* = 3–7 independent primary cultures.EGO enrichment analysis of differentially expressed genes between siLemd2‐ and scrambled siRNA‐transfected Bic‐treated cultures.FVenn diagram illustrating the overlap between the differentially expressed genes (adjusted *P*‐value < 0.05, 0.3 FC cut‐off) in *siLemd2*‐ vs scrambled siRNA‐transfected cultures, SATB2‐deficient vs wild‐type P0 cortices (Fischer’s exact test, *P*‐value < 1E‐15, OR = 2.93), and *Satb2^CamkCre^* vs *Satb2* floxed adult cortices (Fischer’s exact test, *P*‐value < 1E‐15, OR = 3.54).GVenn diagram of SATB2‐bound LEMD2‐regulated genes specific to or shared by the differentially expressed genes in *Satb2* ^NesCre^ cortical neurons (Fischer’s exact test, *P*‐value < 1E‐15, OR = 3.059), P0 cortex (Fischer’s exact test, *P*‐value < 1E‐15, OR = 2.627), and adult cortex (Fischer’s exact test, *P*‐value < 1E‐15, OR = 2.962).HVenn diagram showing the overlap between rapid and delayed PRG (Tyssowski *et al*, 2018) and the genes down‐regulated in Bic‐stimulated *Satb2^NesCre^* CKO (Fischer’s exact test, *P*‐value < 1E‐15, OR = 9.99) and *siLemd2*‐silenced cultures (Fischer’s exact test, *P*‐value < 1E‐15, OR = 10.17).

**Figure 4:**
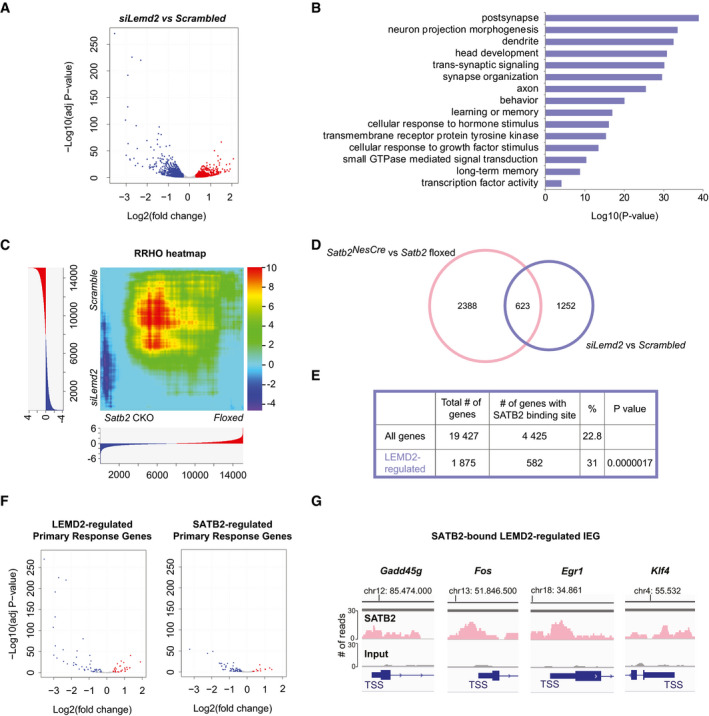
LEMD2 and SATB2 regulate neuronal gene transcription in active neurons Primary cortical Bic‐treated neurons transfected with either scrambled siRNA (*Scrambled*) or siRNA against *Lemd2* (*siLemd2*) were subjected to RNA‐seq. “Volcano plot” of statistical significance against Log2 fold change between *siLemd2‐* and scrambled siRNA‐transfected cultures. The differentially expressed genes are indicated in red (adjusted *P‐*value < 0.05, Log_2_fold change > 0.3) and blue (adjusted *P‐*value < 0.05, Log_2_fold change < −0.3), *n* = 3 independent primary cultures.GO enrichment analysis of the differentially expressed genes between *siLemd2‐* and scrambled siRNA‐transfected cultures.Rank–rank hypergeometric overlap (RRHO) heatmap comparing the global gene expression signatures of *Satb2* ^CamkCre^ vs floxed cortical cultures (*n* = 7) and *siLemd2* vs scrambled siRNA‐transfected cultures (*n* = 3). For each dataset, all expressed genes (gene counts higher than 10) were ranked by their differential expression *P*‐values and effect size direction. The significance of the overlap between the two gene lists is plotted as −log_10_ transformed hypergeometric test *P‐*values corrected for multiple testing by Benjamini and Yekutieli method. The range of the *P*‐values is indicated in the color scale bar.Venn diagram illustrating the overlap between the differentially expressed genes in *siLemd2‐* vs scrambled siRNA‐transfected Bic‐treated cortical cultures and *Satb2* ^CamkCre^ vs *Satb2* floxed primary Bic‐treated cortical cultures (Fisher’s exact test, *P*‐value < 10E‐16, OR = 3.159).Enrichment analysis for SATB2‐bound genes in the LEMD2‐regulated significant gene list (Fischer’s exact test, *P*‐value < 10E‐10, OR = 1.6).“Volcano plots” of statistical significance against Log2 fold change, depicting the PRGs (Tyssowski *et al*, 2018) that were differentially expressed in Bic‐treated *siLemd2‐* vs scrambled siRNA‐transfected cultures (left panel) and Bic‐treated *Satb2* ^CamkCre^ vs *Satb2* floxed primary cortical cultures (right panel). The down‐regulated PRGs are indicated in blue (adjusted *P‐*value < 0.05, Log_2_fold change < −0.3), and the up‐regulated (adjusted *P‐*value < 0.05, Log_2_fold change > 0.3) are shown in red.Examples of IEGs with a SATB2 peak at their proximal promoter that were down‐regulated in both Bic‐treated *Lemd2* knockdown and *Satb2* knockout cultures.

The lamin components of A‐ and B‐type chromatin tethers, i.e. Lamin A/C and Lamin B1, affect developmental gene expression (Solovei *et al*, 2013; Gigante *et al*, 2017). Evidence for a direct role of the INM tether components, e.g. LEM domain‐containing proteins, in gene regulation is currently lacking. We, therefore, investigated whether LEMD2, a likely candidate for the chromatin‐binding mediator of the A tether, affects neuronal gene expression. We compared the transcriptomes of LEMD2‐depleted vs scrambled siRNA‐transfected cortical neurons at two different neuronal activity states: an active state brought about by 1‐h treatment with Bic and an inactive/moderately active state caused by suppression of neuronal activity with the AMPA receptor blocker NBQX for 4 h. As a control experiment for the two treatments, we compared the transcriptomes of Bic‐ vs NBQX‐treated *Satb2* floxed cultures by genome‐wide RNA‐seq. This comparison validated the expected activity‐dependent gene expression changes in our *in vitro* neuronal culture model (Fig EV4A). Since SATB2‐LEMD2‐dependent changes in nuclear shape occur upon Bic treatment (see above), we first tested the effect of LEMD2 depletion in Bic‐treated, synaptically active cultures. Differential expression analysis using DEseq2 (Love *et al*, 2014) revealed a strong effect on gene expression in *Lemd2* knockdown vs control cultures: 1,105 genes were significantly down‐regulated, and 770 genes were up‐regulated (adjusted *P*‐value < 0.05, log_2_FC threshold = 0.3; Fig 4A). Parallel transcriptome profiling of *siLemd2*‐ vs scrambled siRNA‐transfected NBQX‐treated neurons revealed a much weaker effect on gene expression (Fig EV4B), indicating that LEMD2 is particularly relevant for gene regulation in active neurons. Gene ontology (GO) analysis of the differentially expressed genes in Bic‐stimulated cultures demonstrated highly significant overrepresentation of several GO categories (Fig 4B), among them “cellular response to hormone stimulus”. Of note, this GO category is comprised of immediate early response (IEG) genes known to be induced in response to neuronal activity (Yap & Greenberg, 2018). Importantly, IEGs were identified as down‐regulated also in moderately active NBQX‐treated *siLemd2*‐silenced neurons (Table EV1), thus corroborating the effect of LEMD2 depletion on IEG transcription in neurons. In addition, a large cohort of transcription factors with established roles in cortex development was also identified as dysregulated in *Lemd2* knockdown cultures. We observed a significant overlap (hypergeometric *P*‐value = 0.0009, Fischer’s exact test, OR = 1.8) between the genes deregulated in LEMD2‐depleted Bic‐stimulated neurons and the genes encoding deep or upper layer projection neuron‐specific transcription factors (Heavner *et al*, 2020).

To examine whether LEMD2 and SATB2 exert similar effects on gene expression in cortical neurons, we compared LEMD2‐ and SATB2‐dependent transcriptome changes under identical culture conditions. In Bic‐treated cultures derived from *Satb2^NesCre^* mice, the expression of 1,372 and 1,639 genes was decreased and increased, respectively (adjusted P‐value < 0.05, log_2_FC threshold = 0.3 (Fig EV4C). In NBQX‐treated cultures, the effect of *Satb2* ablation was similarly strong: 1,676 and 1,844 genes were down‐ and up‐regulated, respectively (Fig EV4D). In Bic‐treated, synaptically active cultures, the comparison of the global gene expression signatures upon *Lemd2* knockdown and *Satb2* ablation using the threshold‐free rank–rank hypergeometric overlap (RRHO) analysis (Plaisier *et al*, 2010) revealed statistically significant overlap (hypergeometric *P*‐value = 1 × 10^−15^) (Fig 4C) between the two gene sets.

A highly significant overlap was also observed when we compared the lists of LEMD2‐ and SATB2‐regulated genes that were generated by applying fold change and adjusted *P*‐value cut‐offs (Fig 4D).

Furthermore, LEMD2 gene list significantly overlapped with previously described sets of dysregulated genes in SATB2‐deficient *ex vivo* neonatal (McKenna *et al*, 2015) and adult cortices (Cera *et al*, 2019) (Fig EV4D). Functional GO annotation revealed several shared overrepresented GO categories between the LEMD2 and SATB2 gene lists, among them “cellular response to hormone stimulus”, “head development”, and “trans‐synaptic signaling” (Fig EV4E). To gain insights into the potential mechanisms of gene coregulation by SATB2 and LEMD2, we mapped SATB2 ChIP‐seq peaks located within proximal promoters (Jaitner *et al*, 2016) to the list of LEMD2‐regulated genes (Fig 4E). This analysis revealed a significant enrichment of genes containing a SATB2 binding site near their transcriptional start site (TSS) within the LEMD2‐regulated genes (Fig 4E, Table EV2). LEMD2‐regulated genes with a SATB2 peak in their promoter were significantly enriched in all available datasets describing SATB2‐regulated genes in adult cortex, neonatal cortex, and cortical cultures (Fig EV4D).

Remarkably, among the strongest down‐regulated genes in Bic‐stimulated LEMD2‐depleted neurons were IEGs. For example, 18 out of 19 previously described rapid primary response genes (PRG) activated by sustained neuronal activity (Tyssowski *et al*, 2018), and 32 out of 116 delayed PRG were down‐regulated in Bic‐stimulated *Lemd2* knockdown cultures compared to controls (Fig 4F and Table EV3). Activity‐regulated genes (Tyssowski *et al*, 2018) were also enriched among the genes down‐regulated in Bic‐treated *Satb2* knockout cultures (Fig 4F and Table EV3). Out of 135 rapid and delayed PRG (Tyssowski *et al*, 2018), 50 were down‐regulated in Bic‐treated LEMD2‐depleted cultures, 58 in Bic‐treated *Satb2* knockout cultures, and 26 in both (Fig EV4H and Table EV3). Likewise, out of 103 early response genes reported to be induced in excitatory and inhibitory neurons by 1‐h light stimulation of the visual cortex (Hrvatin *et al*, 2018), 53 were down‐regulated in Bic‐treated LEMD2‐depleted cultures (Fischer’s exact test, *P*‐value < 1E‐15, OR = 18.41), 41 were down‐regulated in Bic‐treated *Satb2* knockout cultures (Fischer’s exact test, *P*‐value < 1E‐10, OR = 8.94), and 31 were down‐regulated in both (Table EV3). These results indicate that both LEMD2 depletion and *Satb2* ablation profoundly affect activity‐dependent gene expression in cortical neurons in a highly overlapping manner. Intriguingly, many of these activity‐regulated genes, both early and late‐response genes, contain a SATB2‐binding site within their promoter (Fig 4G) indicating cooperation between the two proteins in the regulation of the activity‐driven IEG response in neurons.

### *In vivo* nuclear plasticity and cFos expression are altered in CA1 pyramidal neurons of SATB2‐deficient mice after exposure to novel environment

**Figure 5:**
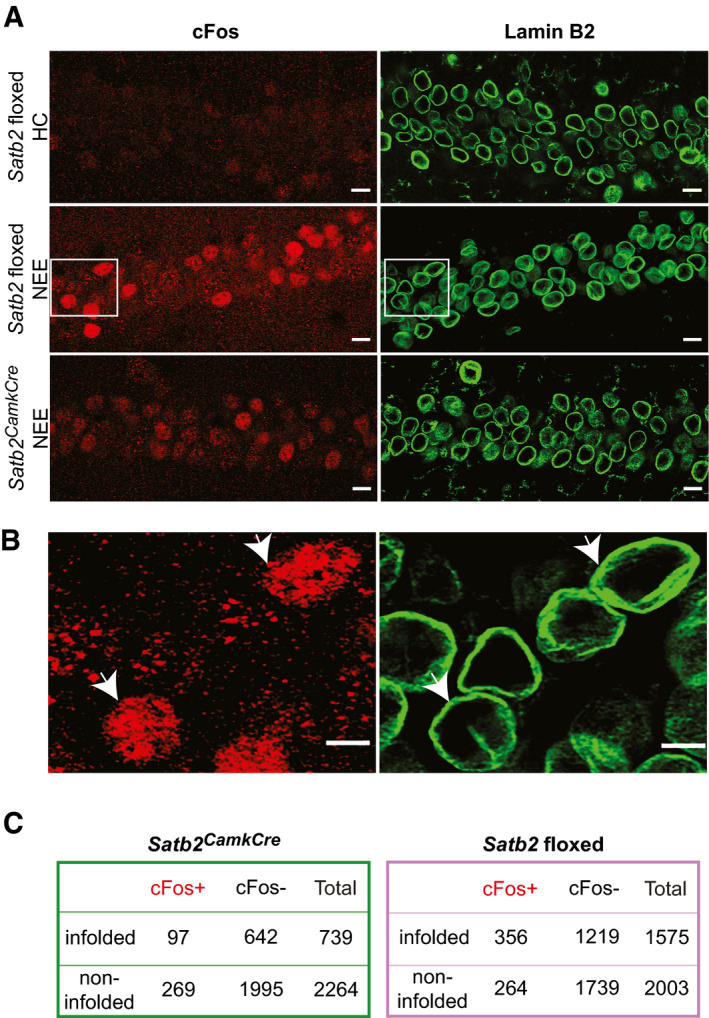
NEE‐activated cFos‐positive neurons are more likely than non‐active neurons to bear nuclear infoldings in *Satb2* floxed but not in *Satb2^CamCre^* mice Representative confocal images (Z‐axis projection) of CA1 pyramidal neurons of: (top panel) *Satb2* floxed mice (*n* = 3) maintained in their home cages (HC), (middle panel) *Satb2* floxed mice (*n* = 4) subjected to NEE for 1.5 h, (bottom panel) *Satb2^CamkCre^* mice (*n* = 5) exposed to NE for 1.5 h. Brain sections were stained for cFos and Lamin B2. Scale bars: 10 μm.Higher magnification views of boxed areas in (A) (middle panel). Arrows show examples of infolded, cFos‐positive nuclei of the CA1 field of *Satb2* floxed mice subjected to NEE. Scale bars: 5 μm.2 × 2 contingency tables showing the number of analyzed CA1 pyramidal neuron nuclei grouped by category: cFos^+^/cFos^−^, infolded/non‐infolded in *Satb2* CKO mice (left) and *Satb2* floxed mice (right). In floxed animals exposed to novel environment, cFos‐positive nuclei were more likely than cFos‐negative nuclei to bear infoldings, chi‐square test of independence, χ^2^ (1, *N* = 3578) = 54.649, *P* < 0.00001. No significant relationship between cFos expression and nuclear infoldings was identified for the CA1 neurons of *Satb2^CamkCre^* mice (χ^2^ (1, *N* = 3003) = 0.80, *P* = 0.3693). Source data are available online for this figure.

To examine the correlation between SATB2‐dependent nuclear shape plasticity and IEG expression *in vivo,* we used the novel environment exposure (NEE) paradigm, i.e. a short‐term exposure of animals to novel and rich spatial context. NEE causes activation of distinct neuronal ensembles throughout the hippocampus (VanElzakker *et al*, 2008), thus allowing for simultaneous analysis of both activated and inactive/baseline population neurons in the same animal. Immunostaining for the IEG cFos was used to identify activated neurons. In line with previous observations (Jaeger *et al*, 2018), the percentage of cFos‐positive nuclei in the CA1 hippocampal field of Satb2 floxed adult mice increased from 2.5 ± 1.2% (SD) in home cage condition to 18.9 ± 2.5% (SD) following NEE (Fig 5A). A significantly lower number of cFos‐positive CA1 neurons was observed in SATB2‐deficient mice after NEE (12.27 ± 2.9% [SD], unpaired *t*‐test, *t*(7) = 3.58, *P* = 0.009; Fig 5A). Furthermore, a chi‐square test of independence revealed a significant relationship between the activity state of CA1 neurons measured by cFos immunoreactivity and the presence of nuclear infoldings revealed by Lamin B2 staining (Fig 5B and C). In floxed animals that had undergone NEE, cFos‐positive nuclei were more likely than cFos‐negative nuclei to bear infoldings (Fig 5C). By contrast, there was no significant relationship between cFos expression and nuclear infoldings in the CA1 neurons of *Satb2^CamkCre^* mice after NEE (Fig 5C). In addition, similar to the data obtained in naïve animals (Fig 2F), there were fewer infolded nuclei in the CA1 field of novelty‐exposed *Satb2^CamkCre^* mice compared to floxed controls (23.92 ± 2.9% [SD] vs 43.59 ± 5.09% [SD],unpaired *t*‐test *t*(8) = 7.48, *P* = 0.00007). Therefore, we conclude that the structural plasticity of neuronal nuclei that manifests as deep invaginations of the nuclear envelope is a correlate of cFos expression triggered by natural sensory stimulation and that SATB2 determines both activity‐dependent nuclear envelope plasticity and cFos induction *in vivo* upon NEE.

### LEMD2‐regulated genes in cortical neurons are relevant for cognition and schizophrenia

**Figure 6:**
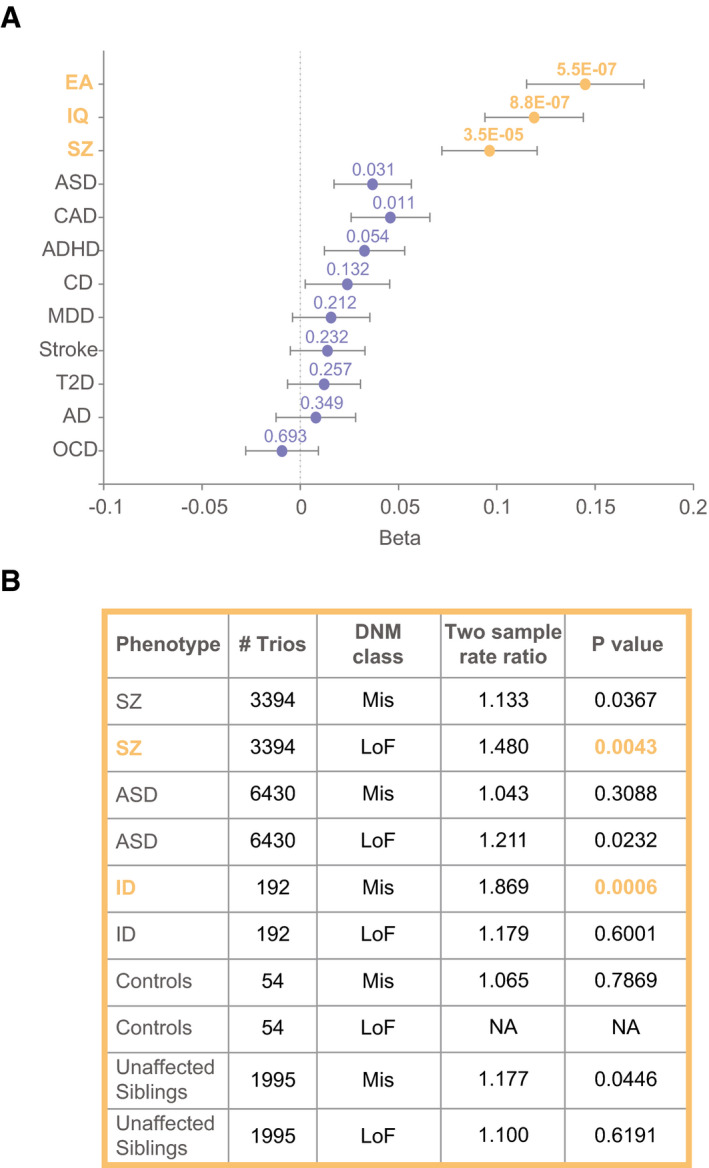
LEMD2‐regulated genes in cortical neurons contribute to cognitive function and risk of neurodevelopmental disorders MAGMA gene set analysis of the differentially expressed genes upon LEMD2 depletion in cortical neurons using the summary statistics from multiple GWAS datasets. Phenotypes are listed on the *y*‐axis, EA (educational attainment), IQ (cognitive ability/human intelligence), SZ (schizophrenia), ASD (autism spectrum disorder), CAD (coronary artery disease), ADHD, (attention‐deficit/hyperactivity disorder), CD (Crohn’s disease), MDD (major depressive disorder), Stroke, T2D (type 2 diabetes), AD (Alzheimer’s disease), and OSD (obsessive–compulsive disorder). *P*‐values are shown above each data point, which represent beta values (*x*‐axis). Horizontal bars indicate error bars (SE). *P*‐values in orange survived Bonferroni multiple test correction of *P* < 0.0125 for the four tests (IQ, EA, ASD, and, SZ). Data also supplied in Table EV4.Analysis of LEMD2‐regulated genes using data on *de novo* mutations. LEMD2 gene set was enriched for LoF *de novo* mutations reported in schizophrenia (SZ) and Mis *de novo*, mutations reported in intellectual disability (ID); *P*‐values in orange survived Bonferroni multiple test correction for the nine tests. Data also supplied in Table EV5.

We have recently demonstrated that the differentially expressed genes in the developing neocortex of *Satb2* mutants are enriched for genes with human orthologues associated with schizophrenia (SZ) and educational attainment (EA) and for genes harboring *de novo* variants reported in autism spectrum disorder (ASD) and intellectual disability (ID) (Whitton *et al*, 2018). Given the highly significant overlap in the transcriptional profiles of SATB2‐deficient and LEMD2‐depleted cortical neurons, we set out to investigate whether LEMD2‐regulated gene set contributes to human psychiatric disorders and cognitive phenotypes. First, we tested whether common genetic variation in human orthologues of the genes differentially expressed upon *Lemd2* knockdown is associated with ASD, SZ, EA, and cognitive ability/human intelligence (IQ) by gene set analysis of recent GWAS data (Lee *et al*, 2018; Pardiñas *et al*, 2018; Savage *et al*, 2018; Grove *et al*, 2019). The LEMD2 gene set was enriched for genes associated with EA, IQ, and SZ (*P*‐values less than the multiple test correction of *P* < 0.0125), but not ASD (Fig 6A, Table EV4). To examine whether the enrichment we detect for EA, IQ, and SZ is a property of polygenic phenotypes in general, we obtained GWAS summary statistics for eight phenotypes (Franke *et al*, 2010; Schunkert *et al*, 2011; Traylor *et al*, 2012; Lambert *et al*, 2013; Mahajan *et al*, 2014; Liu *et al*, 2015; International Obsessive Compulsive Disorder Foundation Genetics Collaborative (IOCDF‐GC) and OCD Collaborative Genetics Association Studies (OCGAS), 2018; Stahl *et al*, 2019; Wray *et al*, 2018; Demontis *et al*, 2019) and we tested the LEMD2 gene set for enrichment in each one. These were childhood‐onset psychiatric disorders, other brain‐related diseases, and non‐brain‐related diseases. No enrichment was detected for any of the eight phenotypes tested (Fig 6A, Table EV4). It is possible that the detected enrichment for EA, IQ, and SZ is due to the LEMD2‐regulated genes representing a set of brain‐expressed genes, which are major contributors to higher cognitive functions. Importantly, the enrichment for genes associated with EA, IQ, and SZ was robust to the inclusion in the analysis of both “brain‐expressed” (*n* = 14,243) and “brain‐elevated” (*n* = 1,424) gene sets as covariates (Table EV4).

Next, we examined whether the LEMD2 gene set was enriched for *de novo* mutations reported in trios‐based studies of ASD, ID, and SZ (Genovese *et al*, 2016; Howrigan *et al*, 2020; Rees *et al*, 2020). For each phenotype, we tested missense (Mis) and loss‐of‐function (LoF) mutations, plus synonymous mutations (Syn) as a control in the ASD, ID, and SZ datasets, and analyzed the control datasets. We found that LEMD2‐regulated genes in cortical neurons were enriched for LoF mutations reported in SZ patients and for Mis mutations reported in ID patients (Fig 6B, *P*‐values less than the multiple test correction of *P* < 0.0056). Analysis of Syn mutations, which are unlikely to be pathogenic in the three disorders, did not reveal significant enrichment. In an additional control measure, both control trios and trios made from unaffected siblings showed no enrichment for *de novo* mutations in the LEMD2 gene set.

Our data reveal a contribution of LEMD2‐regulated genes to human cognitive function and risk of schizophrenia and uncover a previously unexpected overlap of the human phenotypes that converge on gene sets controlled by SATB2 and LEMD2 in pyramidal neuron.

## Discussion

Our study provides evidence that cooperation between the chromatin organizer SATB2, the integral INM protein LEMD2, and the ESCRT‐III/VPS4 membrane remodeling machinery determines plastic changes in nuclear envelope geometry in response to action potential bursting. Our transcriptome analyses in *Satb2* knockout and LEMD2‐depleted cultures correlate these changes in nuclear shape with regulation of the IEG response upon neuronal activation. Furthermore, we demonstrate that *in vivo* cFos expression, triggered in hippocampal neuronal ensembles by natural sensory stimuli, correlates with the changes in nuclear morphology in a SATB2‐dependent manner. Taken together, our findings suggest that formation of an excitatory neuron‐specific SATB2/LEMD2‐containing chromatin tether at the inner nuclear membrane orchestrates activity‐dependent gene expression in pyramidal neurons. Consistent with this hypothesis, human genetic data reveal a contribution of both SATB2‐ and LEMD2‐regulated gene sets to human cognitive function and the risk of schizophrenia.

### SATB2 interactions with LEMD2 and ESCRT‐III complex link activity‐induced nuclear membrane infolding to IEG response

Neuronal activity causes distinct changes in nuclear morphology (Wittmann *et al*, 2009). The precise mechanisms driving activity‐dependent rearrangements in neuronal nuclear structure are unknown. By using gain‐ and loss‐of‐function assays *in vivo* and *in vitro*, we demonstrate that SATB2 is not only required for the dynamic infoldings/invaginations of the nuclear envelope following synaptic activity, but it is also sufficient to induce nuclear membrane invaginations upon overexpression in primary hippocampal neurons. The functional importance of activity‐induced nuclear envelope invaginations is currently poorly understood. It has been proposed that these membrane invaginations generate microdomains of enhanced calcium signaling in the nucleus and increase the surface area of the nuclear envelope (Wittmann *et al*, 2009; Wiegert & Bading, 2011). Our data suggest chromatin tethering and consequently gene regulation as additional functions. We show that activity‐dependent nuclear envelope remodeling in cortical neurons is an active process requiring not only LEMD2 but also the enzymatic activity of the AAA‐ATPase VPS4. Consistent with our findings in primary neurons, LEMD2 has been shown to recruit components of the ESCRT‐III complex to the nuclear envelope in non‐neuronal cells (Olmos *et al*, 2015; Vietri *et al*, 2015; Webster *et al*, 2016; Gu *et al*, 2017). Notably, stable association of LEMD2 with CHMP7, a nucleator of ESCRT‐III at the inner nuclear membrane (Vietri *et al*, 2015; Olmos *et al*, 2016), can cause polymerization of ESCRT‐III complex at the INM, followed by dramatic membrane deformations and local DNA torsional stress (Willan *et al*, 2019; Vietri *et al*, 2020). Thus, it is possible that SATB2/LEMD2/ESCRT‐III/VPS4‐dependent nuclear infoldings formed upon AP bursting, represent a mechanism, whereby topological constraints to IEG expression are resolved enabling transcriptional activation (Madabhushi *et al*, 2015). Consistent with this hypothesis, we observed an impaired IEG response upon both SATB2 knockout and LEMD2 depletion in Bic‐stimulated primary cortical neurons, as well as binding of SATB2 to promoters of IEGs.

### LEMD2 as a dynamic chromatin tether implicated in gene regulation

Cell type‐specific roles of INM proteins are currently poorly understood (Brachner & Foisner, 2011). In non‐neuronal cells, LEMD2 has been implicated in maintaining normal nuclear morphology (Ulbert *et al*, 2006; Abdelfatah *et al*, 2019; Marbach *et al*, 2019). Separate lines of evidence point to a specific role in tethering chromatin to the nuclear envelope (Ikegami *et al*, 2010; Pieper *et al*, 2020; Barrales *et al*, 2016). Here, we demonstrate such a dual function of LEMD2 in cortical neurons, serving both as a regulator of nuclear envelope remodeling and of gene transcription. We hypothesize that physical interaction of LEMD2 with ESCRT‐III and SATB2 establishes an excitatory neuron‐specific chromatin‐NL tether, affecting both nuclear geometry and gene expression simultaneously. Perhaps a recently identified phase separating property of LEMD2 (von Appen *et al*, 2020) also contributes to the formation of nuclear infolding function. We note that the subset of SATB2‐LEMD2 coregulated genes varies between neonatal and adult cortex. Likewise, the set of SATB2‐LEMD2 coregulated genes with a SATB2 binding site at their promoter‐also changes between different developmental stages. These findings suggest a dynamic nature of the hypothetical SATB2‐LEMD2‐containing tether that is likely to affect different sets of genes depending on the cellular context. We also identified many LEMD2‐regulated genes that are not affected by SATB2 ablation. These results suggest an important role of LEMD2 as a gene regulator in cortical neuron development and/or maturation that goes beyond interaction with SATB2. LEMD2, which is ubiquitously expressed, seems to represent a versatile platform for protein–protein interactions, potentially recruiting other transcriptional regulators or epigenetic modifiers to the NL.

A prominent group of genes commonly affected by SATB2 ablation or LEMD2 depletion is IEGs described in the neuronal response to stimulation (Hrvatin *et al*, 2018; Tyssowski *et al*, 2018). We found that in both SATB2‐deficient and LEMD2‐depleted cultures, the expression of multiple IEG is reduced not only under strongly activated but also under moderately active/inhibited conditions. While the effect of SATB2 ablation was similarly strong in activated and silenced cultures, LEMD2 knockdown affected weakly the IEG expression in silenced cultures and much more prominently upon activation. This indicates that gene regulatory mechanisms linked to LEMD2 might themselves be subjected to regulation by neuronal activation. Activity‐dependent spatial relocation of some IEGs to the nuclear interior (Walczak *et al*, 2013) or to transcription factories (Crepaldi *et al*, 2013) has been described. Therefore, it would be important in the future to test whether translocation of these genes also occurs relative to the infoldings of the nuclear envelope that appear upon neuronal activation. However, the complex changes in nuclear geometry that accompany nuclear infolding formation currently preclude testing of this hypothesis until relevant parameters for distance measurements in these complex infolded three‐dimensional shapes are established.

### Similar to SATB2, LEMD2‐regulated genes are associated with human cognitive ability

To provide further support for a contribution of LEMD2‐regulated genes to brain development and function, we tested their association with human neuropsychiatric disease and cognitive phenotypes. In line with our hypothesis for an important role of LEMD2 in cortex development, the gene set affected by *Lemd2* knockdown was enriched for genes harboring *de novo* mutations reported in ID, a severe early age‐onset neurodevelopmental disorder, and in SZ, which is also considered part of the neurodevelopmental continuum (Owen & O’Donovan, 2017). Importantly, the analysis based on common GWAS variants showed enrichment for genes associated with SZ and general cognitive ability. Cognitive dysfunction is a core symptom of SZ. SATB2‐regulated and targeted genes (Whitton *et al*, 2018) and the genes encoding SATB2 binding partners (Cera *et al*, 2019) are also enriched for common variants associated with cognitive function. Thus, based on the observation that human cognitive phenotypes converge on both SATB2 and LEMD2 gene‐sets, it appears likely that activity‐dependent or ‐independent gene regulation by LEMD2‐SATB2‐containing chromatin tethers is important for human cognitive ability. In a broader context, the identified enrichment for genes associated with IQ, and genes contributing to ID, and SZ in the LEMD2 gene set highlights previously undescribed contributions of NL‐directed transcriptional regulation to the etiology of human neuropsychiatric disorders.

## Materials and Methods

### Animals

*Satb2* conditional mutants, i.e. adult forebrain, pyramidal neuron‐specific, *Satb2^flx^* ^/^ *^flx^::Camk2a‐Cre*, and neonatal pan‐neuronal, S*atb2^flx^* ^/^ *^flx^::Nes‐Cre,* have been previously described (Jaitner *et al*, 2016; Cera *et al*, 2019). All experimental procedures have been approved by the Austrian Animal Experimentation Ethics Board.

### Antibodies

The following primary antibodies were used: α‐BAF (PU38143, a generous gift from T. Haraguchi), α‐LEMD2 (HPA017340, Sigma), α‐SATB2 (ab92446, Abcam), α‐V5 epitope tag (R960‐25, Thermo Fisher Scientific), α‐Lamin B2 (33‐2100, Thermo Fisher Scientific), α‐Fos (226004, Synaptic Systems), α‐Lamin B1 (ab16048, Abcam), α‐VPS4 (E‐8) (sc‐133122, Santa Cruz), α‐phospho‐MSK1 (Thr581) (#9595, New England Biolabs), α‐ERK2 (C‐14) (sc‐154, Santa Cruz), α‐GAPDH (MAB374, Millipore), and α‐beta‐III Tubulin (NB100‐1612, Novus Biologicals).

Secondary antibodies were as follows: goat anti‐mouse Alexa‐488 (A11001, ab150117), goat anti‐mouse Alexa‐555 (A21422), donkey anti‐mouse Alexa‐488 (A21202), goat anti‐rabbit Alexa‐555 (A21428), goat anti‐rabbit Alexa‐488 (ab150081), donkey anti‐rabbit Alexa‐555 (ab150062), and donkey anti‐goat Alexa‐555 (A21432).

### Neuronal cultures

Hippocampi were dissected from C57BL/6J mice, *Satb2^flx^* ^/^ *^flx^::Nes‐Cre* mice or *Satb2^flx^* ^/^ *^flx^* mice at postnatal days P0–P1. Hippocampal tissue was trypsinized and dissociated by trituration. Neurons were plated at a density of 8 × 10^4^ cells/cm^2^ on either 35 mm tissue culture dishes or 18 mm coverslips, previously coated with poly‐l‐ornithine (Sigma) and laminin (Sigma) and cultured as previously described (Jaitner *et al*, 2016).

Dissected cortical tissue from neonatal C57BL/6J, *Satb2^flx^* ^/^ *^flx^::Nes‐Cre* mice or *Satb2^flx^* ^/^ *^flx^* mice was washed with Hank's balanced salt solution without calcium, magnesium, and sodium bicarbonate (Thermo Fisher Scientific) and incubated in prewarmed enzyme solution (Papain 20 Units/ml, Worthington) containing 10 mg/ml DNase I (Sigma) for 10 min at 37°C. Dissociated cortical neurons were plated 9 × 10^4^ cells/cm^2^ in serum‐containing attachment medium (MEM, 10% horse serum, Thermo Fisher Scientific) on plates previously coated with poly‐l‐ornithine (Sigma) and laminin (Sigma). After preculturing for 2 h, MEM was exchanged with Neurobasal A medium, supplemented with B27 (Thermo Fisher Scientific), Glutamax (Thermo Fisher Scientific), and penicillin/streptomycin (Thermo Fisher Scientific). Neurons were cultured for 14 days at 5% CO_2_ and 37°C. Glial cell proliferation was inhibited by adding cytosine arabinoside (5 µM, Sigma) to the culture medium at DIV3. Treatment with bicuculline (Bic, 50 μM, Tocris) or NBQX (10 µM, Tocris) was performed at DIV10 (hippocampal neurons) or DIV14 (cortical neurons).

### siRNA‐ or shRNA‐mediated gene silencing

siRNA‐mediated silencing of *Lemd2* or *Vps4a*/*Vps4b* was carried out using Accell SMARTpool siRNA Reagents (mouse *Lemd2* siRNA—SMARTpool E‐052028‐00‐0020, mouse *Vps4a* siRNA—SMARTpool E‐046156‐00‐0020, and mouse *Vps4b* siRNA—SMARTpool E‐044487‐00‐0020, Horizon). Accell Non‐Targeting siRNA (D‐001910‐10‐50, Horizon) was used as control. Briefly, primary neurons were transfected with siRNA (1 µM) according to the manufacturer's instructions at DIV 6. The transfected cells were used for analysis (immunocytochemistry, immunoblotting, or RNA‐seq) on DIV10 (hippocampal neurons) or DIV14 (cortical neurons).

pAAV‐U6‐hSyn::mCherry.3xFLAG‐WPRE (Grünewald *et al*, 2019) was purchased from Addgene (#120392) and used for cloning of shRNAs targeting *Lemd2*: shRNA1 (5′‐TTGGCTGCTGCACGAACTGTACTTCAAGAGAGTACAGTTCGTGCAGCAGCT‐3′) and shRNA2 (5′–TTGATTGTTGCCGGTCGACTGTTTCAAGAGAACAGTCGACCGGCAACAATT‐3′). Equimolar mixes of pAAV constructs targeting *Lemd2* and the plasmid encoding scrambled siRNA, pAAV‐U6‐scrambled‐hSyn::mCherry.3xFLAG‐WPRE (Addgene, #120395) were packaged at the Charite Viral Core Facility, Berlin, Germany.

### HeLa cell culture and transfections

HeLa cells were grown in DMEM medium supplemented with 10% FBS and 1% penicillin/streptomycin (Thermo Fisher Scientific). Transfection was performed using jetPEI DNA transfection reagent (Polypus) according to manufacturer's instructions.

The following mammalian expression plasmids were used: V5‐tagged *Satb2* full‐length and deletion mutants (pEF‐DEST51‐SATB2^1–733^, pEF‐DEST51‐SATB2^346–733^, pEF‐DEST51‐SATB2^616–733^, pEF‐DEST51‐SATB2^1–249^, pcDNA‐DEST47‐SATB2^1–157^); V5‐tagged LEMD2 and deletion mutants (pDEST51‐LEMD2, pDEST51‐LEMD2*^ΔLEM^*, pDEST51‐LEMD2*^413–503^*), GFP‐tagged VPS4A (peGFP‐VPS4A‐C1), and GFP‐tagged VPS4A dominant‐negative (DN) mutant (peGFP‐VPS4A^E288Q^‐C1). Empty pDEST51 or pEGFP‐C1 plasmids were used as controls. Forty‐eight hours after transfection, total protein lysates were prepared and used for immunoprecipitation or immunocytochemistry.

### Co‐immunoprecipitation

Neonatal cortical tissue was homogenized using a dounce homogenizer in IP lysis buffer (25 mM Tris–HCl pH 7.4, 150 mM NaCl, 1% NP‐40, 1 mM EDTA, 5% glycerol; Pierce). DIV13 cortical cultures were lysed on the culture dish in IP lysis buffer (Pierce). The lysates were incubated for 10 min on ice while shaking, followed by a brief centrifugation at 13,000 *g*. The supernatant was used in immunoprecipitation reactions using the Dynabeads Protein G Immunoprecipitation Kit (Thermo Fisher Scientific) according to the manufacturer’s instructions. Briefly, 50 µl of protein G Dynabeads was coated with 5 µg of anti‐SATB2 antibody (ab92446, Abcam), and beads were mixed with 500 µg of cortical lysate and incubated overnight at 4°C. On the next day, the beads–antibody–protein complexes were washed three times with washing buffer, re‐suspended in 2 × Roti‐Load sample buffer (Roth) for elution, and incubated at 95°C for 5 min. The eluates were separated by SDS–PAGE and used for further immunoblotting analysis.

### Immunoblotting

Total protein lysates were prepared in 2 × Roti‐Load sample buffer (Roth). Western blotting was performed as described previously (Loy *et al*, 2011). Membranes were blocked with 5% milk in TBST (0.1% Tween 20 in TBS) for 1 h and then incubated overnight at 4°C with the corresponding primary antibodies diluted in blocking solution. After incubation with HRP‐coupled secondary antibodies, the blots were incubated with ECL reagent (Bio‐Rad) and developed using the BioRad chemiluminescence detection system (ChemiDoc^™^).

### *In vitro* GST pull‐down assays

SATB2 or LEMD2^395–503^ C‐terminal fragment were cloned into the pGEX‐5X1 vector with a GST tag. All GST hybrid proteins were expressed in *Escherichia coli* (strains: BL21‐DE3‐RIL, Rosetta pLysS). Induction, cell lysis, and affinity purification of hybrid proteins were performed as recommended by the supplier of the pGEX vectors (GE Healthcare). GST hybrid proteins were immobilized on glutathione beads and incubated with lysates from HeLa cells transfected with mammalian expression plasmids encoding V5‐tagged LEMD2 (pDEST51‐LEMD2), V5‐tagged LEMD2*^ΔLEM^*, or V5‐tagged SATB2. Resin‐associated complexes were washed at least four times with the standard lysis buffer (10 mM sodium phosphate pH 7.2, 150 mM NaCl, 0.5% Triton X‐100) and eluted with Laemmli sample buffer (2% SDS, 50 mM Tris–HCl pH 6.8, 0.2 mg/ml bromphenol blue, 0.1 M DTT, 10% (v/v) glycerol). The eluates were analyzed by immunoblotting as described above.

### AAVs, viral transductions, and stereotaxic injections

Primary hippocampal or cortical neurons were transduced with AAVs (AAV8‐hSyn‐EGFP, AAV8‐hSyn‐SATB2, AAV8‐U6‐shLemd2, AAV8‐U6‐scrambled) at multiplicity of infection 1.5 × 10^5^ at DIV4 and used for immunofluorescence analysis at DIV10 and DIV14, respectively.

AAVs were delivered by bilateral stereotaxic injections into the dorsal hippocampus of 12‐week‐old male mice as previously described (Jaitner *et al*, 2016).

### Novel environment exposure

*Satb2^flx^* ^/^ *^flx^::Camk2a‐Cre* and *Satb2^flx^* ^/^ *^flx^* mice (12 weeks old) were individually housed for 3–4 days and handled for 5 min per day before exposing them to novel environment (NE). The NE cage (transparent polycarbonate box, 427 × 287 × 198 mm) contained bedding material and objects with different shapes and colors (tunnels, LEGO objects, huts, fruit). Mice were individually placed in a NE cage for 90 min (NE group) or received no exposure (HC group) before sacrificing them by anesthetic overdose and processing for immunohistochemistry.

### Immunohistochemistry

Mice were anesthetized and transcardially perfused with 4% PFA (w/v) in PBS (pH 7.4). Brains were removed, postfixed for 6 h in 4% PFA, cryo‐protected in 30% (w/v) sucrose at 4°C, and embedded in Tissue‐Tek O.C.T. Compound medium (Sakura Finetek). Free‐floating cryosections (40 µm) were washed three times in Tris‐buffered saline (TBS), permeabilized in 0.3% (v/v) Triton X‐100 in TBS for 5 min, and incubated with blocking solution (10% (v/v) normal serum, 1% (w/v) BSA, 0.3% Triton X‐100 in TBS) for 2 h. Sections were incubated with the corresponding primary antibodies at 4°C for overnight. After three washes in 0.025% Triton X‐100 in TBS, sections were incubated with the corresponding secondary antibodies. Sections were counterstained with Hoechst 33258 (H‐3569, Molecular Probes) for 5 min or with RedDot2 (40061, Biotium) for 20 min. After three washes in TBS, sections were mounted with Roti‐Mount FluorCare mounting medium (Roth).

### Microscopy and image analysis

Nuclear geometry was analyzed in z‐stacks of confocal images using a LSM 510/Axiovert200M microscope (Zeiss) and ZEISS ZEN 2009 Imaging Software. For immunocytochemistry analysis, a Zeiss alpha Plan‐Fluar 100×/1.49 Oil M27 was used and the image size was set to 512 × 512 pixels. Image stacks of 40–60 images (0.2 µm intervals), spanning the entire nucleus, were acquired from randomly selected regions of the coverslips. Maximum intensity projections were created using the NIH ImageJ software, and images were analyzed for infolded nuclei as previously described (Wittmann *et al*, 2009) in a blinded to the experimental condition fashion. A nucleus was considered infolded if carrying one or more invaginations spanning at least 50% of the nuclear diameter. Average phospho‐MSK1 immunoreactivity was measured as absolute eight bit gray values (after background subtraction. Hoechst staining was used to identify nuclei.

For brain section microscopy, a Zeiss Plan‐Apochromat 63×/1.4 Oil DIC objective was used and the image size was set to 1,024 × 1,024 pixels. Image z‐stacks (36 images, 0.32 µm intervals) were taken from the hippocampus of *Satb2* ^flx/flx^::*Camk2a‐Cre* and *Satb2* ^flx/flx^ mice under the same microscope settings. Only nuclei that were fully represented in the z‐stacks were counted by using NIH ImageJ software and analyzed for nuclear infoldings following the same criteria as for primary neurons. To quantify cFos‐positive nuclei, images were background subtracted (3.5 × mean fluorescence of several random regions of interest located outside the CA1 pyramidal cell layer), nuclei in the CA1 pyramidal layer were manually traced in the Lamin B2 channel using ImageJ software, and the mean fluorescence was measured in the corresponding cFos channel. The designation “cFos‐positive” was assigned to the nuclei, which had mean fluorescence values above the 3.5 × background value. Likewise, for the quantification of AAV‐*shLemd2*‐ and AAV‐scrambled shRNA‐transduced CA1 neurons, the traced regions were copied in the RFP/mCherry channel for analysis and the designation “AAV‐transduced” was assigned to cells whose nuclei had mean fluorescence values above the background value. Images were analyzed in a blinded to the experimental condition fashion.

For the acquisition of low magnification images of AAV‐shRNA‐transduced dorsal hippocampus of wild‐type mice, a LSM 980/Airyscan 2 microscope (Zeiss) equipped with Plan‐Apochromat 10×/0.45 M27 objective was used.

### RNA‐seq

RNA‐seq analysis was carried out as previously described (Cera *et al*, 2019). In brief, RNA was isolated from primary cortical neurons using PureLink™ RNA Micro Scale Kit (Thermo Fisher Scientific). Libraries were made according to Illumina standard protocols (TruSeq, Illumina) and sequenced as 50 bp single‐end reads on a HiSeq 2000 or as 150 bp paired‐end reads on a Novaseq 6000 platform according to established procedures. RNA‐seq reads were mapped to mouse reference genome (mm10) with STAR aligner (Dobin *et al*, 2013). Read counts were obtained using featureCounts (Liao *et al*, 2014) and normalized using the normalization algorithms of DESeq2 (Love *et al*, 2014). Differential gene expression analysis was performed in DESeq2, accounting for hidden batch effects by the removal of unwanted variation (RUVr) method (Risso *et al*, 2014). A threshold cut‐off of adjusted (Benjamini–Hochberg) *P*‐value < 0.05 and log_2_ fold change (FC) < −0.3 or > 0.3 was applied.

The gene expression profiles of *Satb2* knockout vs *Satb2* floxed cortical neurons and *Lemd2* knockdown vs control cultures were compared by means of a rank–rank hypergeometric overlap (RRHO) analysis (Plaisier *et al*, 2010). RRHO heat maps that graphically visualize correlations between two expression profiles were generated at http://systems.crump.ucla.edu/rankrank/.

### Functional annotation

Pathway and process enrichment analysis was carried out by using the Metascape bioinformatics tool (http://metascape.org) with the following ontology sources: KEGG Pathway, GO Biological Processes, GO Cellular Components, GO Molecular Functions, and CORUM. All genes in the genome were used as the enrichment background. Terms with a *P*‐value < 0.01, a minimum count of 3, and an enrichment factor > 1.5 (the ratio between the observed counts and the counts expected by chance) were collected and grouped into clusters based on their membership similarities. *P*‐values were calculated based on the accumulative hypergeometric distribution, and *q*‐values were calculated using the Benjamini–Hochberg procedure to account for multiple testing. Kappa scores were used as the similarity metric when performing hierarchical clustering on the enriched terms, and sub‐trees with a similarity of > 0.3 were considered a cluster. The most statistically significant term within a cluster was chosen to represent the cluster.

### GWAS data

The LEMD2 gene set (adjusted *P*‐value < 0.05) was tested for enrichment of genes associated with different neurodevelopmental phenotypes using GWAS summary statistics for SZ (Pardiñas *et al*, 2018), ASD (Grove *et al*, 2019), IQ (Savage *et al*, 2018), and EA (Lee *et al*, 2018). For control purposes, we tested for enrichment using GWAS summary statistics for eight other phenotypes: attention‐deficit/hyperactivity disorder (Demontis *et al*, 2019), coronary artery disease (Schunkert *et al*, 2011), major depressive disorder (Wray *et al*, 2018), obsessive–compulsive disorder (International Obsessive Compulsive Disorder Foundation Genetics Collaborative (IOCDF‐GC) and OCD Collaborative Genetics Association Studies (OCGAS), 2018), Alzheimer’s disease (Lambert *et al*, 2013), Crohn’s disease (Franke *et al*, 2010), stroke (Traylor *et al*, 2012), and type 2 diabetes (Mahajan *et al*, 2014).

### MAGMA gene set analysis

A gene set analysis (GSA) is a statistical method for simultaneously analyzing multiple genetic markers in order to determine their joint effect. We performed GSA using MAGMA (de Leeuw *et al*, 2015) and summary statistics from various GWAS. An analysis involved three steps. First, in the annotation step we mapped SNPs with available GWAS results on to genes (GRCh37/hg19 start‐stop coordinates ± 20 kb). Second, in the gene analysis step we computed gene *P*‐values for each GWAS dataset. This gene analysis is based on a multiple linear principal components regression model that accounts for linkage disequilibrium (LD) between SNPs. The European panel of the 1000 Genomes data was used as a reference panel for LD. Third, a competitive GSA based on the gene *P*‐values, also using a regression structure, was used to test whether the genes in a gene set were more strongly associated with either phenotype than other genes in the genome. The MHC region is strongly associated in the SZ GWAS data. This region contains high LD, and the association signal has been attributed to just a small number of independent variants (Sekar *et al*, 2016). However, MAGMA still identifies a very large number of associated genes despite factoring in the LD information. To avoid the excessive number of associated genes biasing the MAGMA GSA, we excluded all genes within the MHC region from our GSA of SZ. MAGMA was chosen because it corrects for LD, gene size, and gene density (potential confounders) and has significantly more power than other GSA tools (de Leeuw *et al*, 2016).

### Enrichment analysis for genes containing de novo mutations

Lists of genes harboring de novo mutations (DNMs) identified in patients with ASD (*n* = 6,430), ID (*n* = 192) and in unaffected siblings (*n* = 1,995) and controls (*n* = 54) based on exome sequencing of trios were sourced from Genovese *et al* (2016) and Satterstrom *et al* (2020). Genes containing DNMs reported in SZ patients (*n* = 3,444) were taken from Howrigan *et al* (2020) and Rees *et al* (2020). DNMs were categorized as synonymous, missense, and loss‐of‐function (included non‐sense, frameshift, and splice site mutations). We tested for enrichment of our gene set in these gene lists using the R package denovolyzeR (Ware *et al*, 2015). For each gene in a gene set, denovolyzeR counts the numbers of observed DNMs and derives the expected number of DNMs in a given population based on the mutability of the gene and the number of trios sequenced (Ware *et al*, 2015). Enrichment of DNMs in a test gene set was investigated using a two‐sample Poisson rate ratio test, using the ratio of observed to expected DNMs in genes outside of the gene set as a background model.

### Statistical analysis

Statistical significance was determined by Student’s *t*‐test, one‐way, or two‐way ANOVA followed by an appropriate *post hoc* test (Tukey, Hochberg, or Bonferroni). Data represent mean ± SEM of at least three independent biological replicates. Although our sample sizes were similar to those reported in previous studies, no statistical methods were used to predetermine sample sizes.

## Authors contributions

PF, AA, IC, CA, MB, and NW performed the experiments. ES, AF, DWM, LW, AL, and GA analyzed the data with contributions from PF, AA, and IC. CA, DT, and SS contributed to the design of the work. GA and GD designed the experiments and wrote the paper.

## Conflict of Interest

The authors declare that they have no conflict of interest.

## Supporting information



Appendix

Expanded View Figures PDF

Table EV1

Table EV2

Table EV3

Table EV4

Table EV5

Source Data for Expanded View

Reviw Process File

Source Data for Figure 1

Source Data for Figure 2

Source Data for Figure 3

Source Data for Figure 5

## Data Availability

The datasets produced in this study are available in the following databases: RNA‐Seq data: Gene Expression Omnibus GSE157375 (https://www.ncbi.nlm.nih.gov/geo/query/acc.cgi?acc=GSE157375).
