# Supplementary material for: SATB2‐LEMD2 interaction links nuclear shape plasticity to regulation of cognition‐related genes
Source: EMBO J. 2020 Dec 15;40(3):e103701. doi: 10.15252/embj.2019103701 (PMC7849313; doi:10.15252/embj.2019103701)
Supplement: Supplementary file 1 — Appendix [file EMBJ-40-e103701-s001.pdf]

Table of Contents:

1. Appendix Figure S1
   Phenotype of Satb2 $^{flx/flx}$ and Satb2^{NesCre}cKO mouse brain

2. Appendix Figure S2
   Coomassie Brilliant Blue Staining of purified GST-hybrid proteins

3. Appendix FIgure S3
   AP bursting increases nuclear phospho-MSK1 immunoreactivity in both *Satb2*$^{flx/flx}$ and *Satb2*^{NesCre} hippocampal neurons

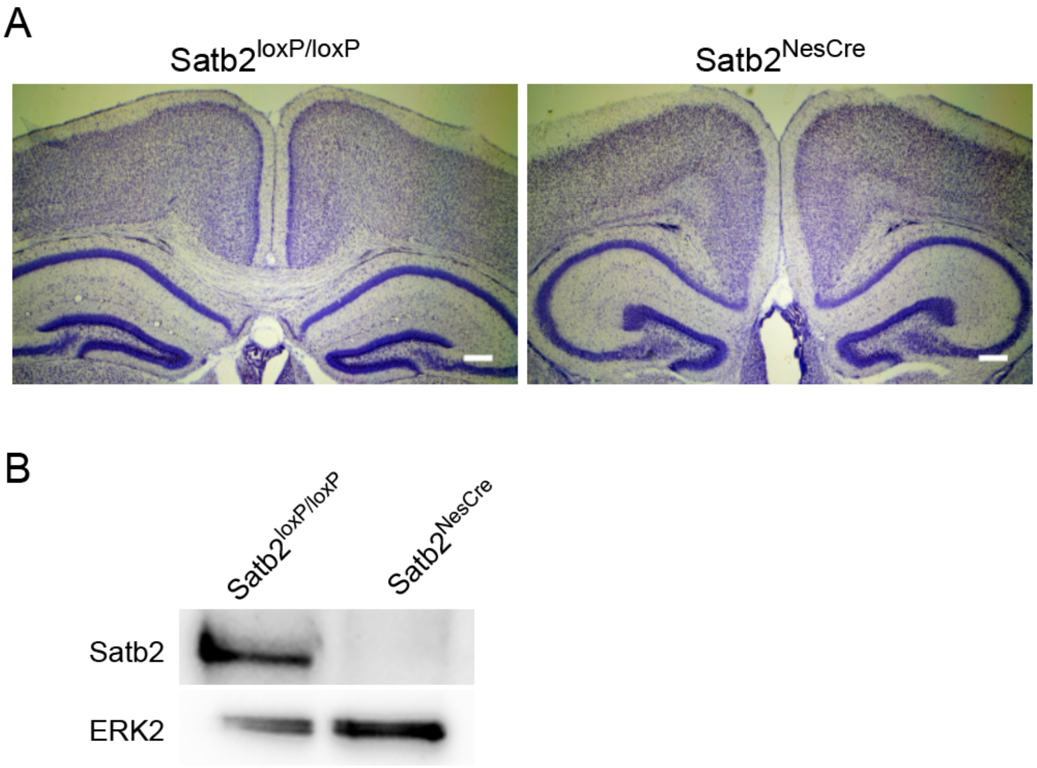

Appendix Figure S1.: Phenotype of Satb2$^{flx/flx}$ and Satb2^{NesCre}cKO mouse brain

A Nissl-staining of coronal brain sections from P15 Satb2$^{flx/flx}$ (left panel) and Satb2^{NesCre} knockout mice (right panel). Satb2^{NesCre} mice (Cera et al., 2019) lack corpus callosum as has been described for Satb2-germ line knockout mutants (Alcamo et al., 2008; Britanova et al., 2008). Scale bar: 200 µm.

B Immunoblotting analysis for Satb2 in DIV7 primary hippocampal cultures derived from Satb2^{NesCre} or Satb2$^{loxP/loxP}$ mice demonstrates absence of detectable Satb2 immunoreactivity in cultures from knockout animals compared to cultures from floxed littermate control mice.

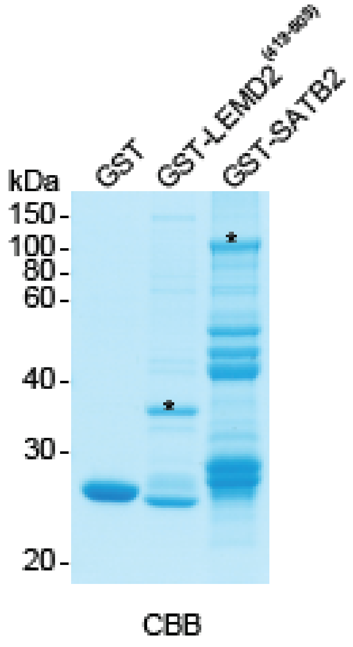

Appendix Figure S2.: Coomassie Brilliant Blue Staining of purified GST-hybrid proteins

Coomassie Brilliant Blue (CCB)-stained gel of protein fractions obtained after affinity purification of GST-hybrid proteins is shown. Asterisks indicate the GST-SATB2 and GST-LEMD2$^{(413\text{-}503)}$ hybrid proteins.

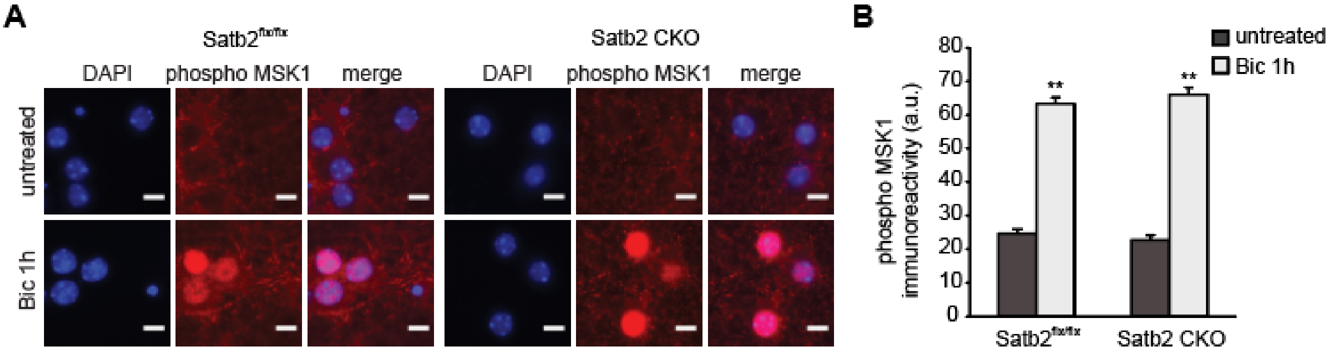

Appendix Figure S3.: **AP bursting increases nuclear phospho-MSK1 immunoreactivity in both Satb2flx/flx and Satb2-defficient hippocampal neurons**

Immunocytochemical analysis of the phosphorylation of MSK1 at threonine 581 following Bic-induced AP bursting for 1 h in primary hippocampal cultures derived from neonatal Satb2$^{flx/flx}$ mice and Satb2$^{flx/flx}$::Nes-Cre mice. The mean nuclear phospho-MSK1 immunoreactivity was measured as absolute 8 bit gray levels. DAPI staining was used to identify nuclei. Representative images **(A)** and the quantitative analysis **(B)** are shown. AP bursting caused a significant increase in the nuclear phospho MSK1 immunoreactivity in both Satb2$^{flx/flx}$ and Satb2$^{flx/flx}$::Nes-Cre cultures, *n* = 3 independent primary cultures, two-way ANOVA; main effect of treatment, $F_{1,8}$ = 46.48, p = 0.0001; main effect of genotype, $F_{1,8}$ = 0.003, p = 0.95, not significant interaction, $F_{1,8}$ = 0.15, p = 0.7081; untreated vs. Bic-treated Satb2$^{flx/flx}$ cultures p = 0.0081; untreated vs. Bic-treated Satb2$^{flx/flx}$::Nes-Cre cultures p = 0.0041, adjustment for multiple comparisons: Tukey's. Number of analyzed nuclei: 532 (Satb2$^{flx/flx}$ cultures, untreated); 461 (Satb2$^{flx/flx}$ cultures, Bic-treated); 520 (Satb2$^{flx/flx}$::Nes-Cre cultures, untreated), 492 (Satb2$^{flx/flx}$::Nes-Cre , Bic-treated). Data are presented as mean ± SEM, **p < 0.01, Scale bar, 10 µm.
