# Supplementary material for: SATB2‐LEMD2 interaction links nuclear shape plasticity to regulation of cognition‐related genes
Source: EMBO J. 2020 Dec 15;40(3):e103701. doi: 10.15252/embj.2019103701 (PMC7849313; doi:10.15252/embj.2019103701)
Supplement: Supplementary file 2 — Expanded View Figures PDF [file EMBJ-40-e103701-s002.pdf]

Expanded View Figures

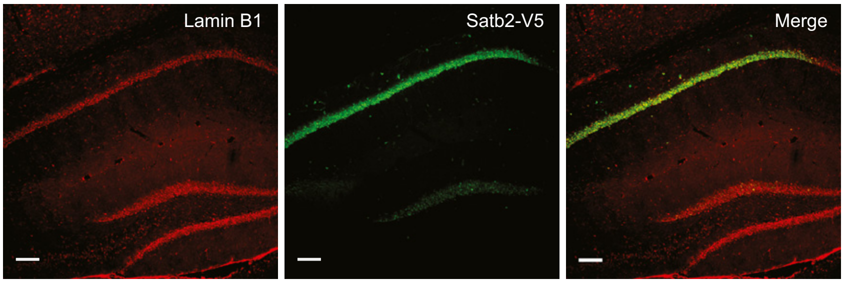

Figure EV1. Re-introduction of V5-tagged SATB2 into the adult dorsal hippocampus of Satb2 conditional mutants.
rAAV8-hSyn-*Satb2-V5* virus was injected into the dorsal hippocampus of *Satb2*^{CamKCre} knockout mice. Lamin B2 staining (left panel) was used to mark cell nuclei in hippocampus. V5 immunoreactivity was used to detect expression of SATB2-V5 in hippocampus (middle panel). Overlay of both pictures (right panel) demonstrates re-expression of SATB2 in CA1-neuronal nuclei in SATB2-deficient mice. Representative images are shown. Scale bars: 100 µm.

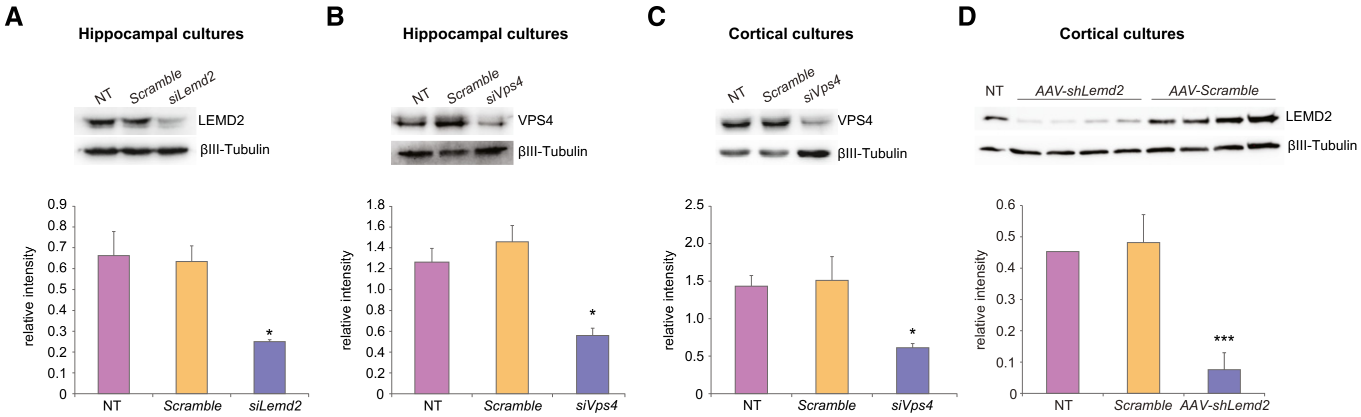

Figure EV2. Gene silencing of *Lemd2*, *Vps4a* and *Vps4b* in primary neuronal cultures.

A Gene silencing of *Lemd2* in hippocampal cultures by siRNA. Upper panel: Representative Western blot for LEMD2 protein in primary hippocampal neurons, *NT* (non-transfected), *Scramble* (transfected with control siRNA), *siLemd2* (transfected with siRNA against *Lemd2*), Lower panel: Western blot quantification of LEMD2 in hippocampal cultures, $n = 4$ independent primary cultures. One-way Welch's ANOVA followed by Tukey *post hoc* test, $F_{2,9} = 16.783$, $P = 0.011$, NT vs *siLemd2* $P = 0.013$, *Scramble* vs *siLemd2* $P = 0.019$. Data are presented as mean ± SEM, *$P < 0.05$ compared to NT.

B Gene silencing of *Vps4* in hippocampal cultures by siRNA. Upper panel: Representative Western blot for VPS4 protein level in primary hippocampal neurons. *NT* (non-transfected), *Scramble* (transfected with control siRNA), *siVps4* (transfected with siRNAs against *Vps4a* and *Vps4b)*. Lower panel: Western blot quantification of VPS4 protein level in primary hippocampal cultures, $n = 3$ independent experiments, one-way Welch's ANOVA followed by Tukey *post hoc* test, $F_{2,6} = 14.226$, $P = 0.027$, NT vs *siVps4* $P = 0.063$, *Scramble* vs *siLemd2* $P = 0.045$. Data are presented as mean ± SEM, *$P < 0.05$ compared to *scramble*.

C Gene silencing of *Vps4* in cortical cultures by siRNA. Upper panel: Representative Western blot for VPS4 protein level in primary cortical neurons. *NT* (non-transfected), *scramble* (transfected with control siRNA), *Vps4* (transfected with siRNA against *Vps4a* and *Vps4b*), Lower panel: Western blot quantification of VPS4 protein level in primary cortical cultures, $n = 3$ independent experiments, one-way ANOVA followed by Tukey *post hoc* test, $F_{2,8} = 9.423$, $P = 0.014$, NT vs *siVps4* $P = 0.041$, *scramble* vs *siVps4* $P = 0.015$. Data are presented as mean ± SEM, *$P < 0.05$ compared to NT.

D Gene silencing of *Lemd2* in cortical cultures by shRNA. Upper panel: Western blot for LEMD2 in primary cortical neurons, *NT* (non-transfected), *Scramble* (transfected with control shRNA), *AAV-shLemd2* (transfected with shRNA against *Lemd2*). Lower panel: Western blot quantification of LEMD2 protein level, $n = 4$ independent experiments, unpaired *t*-test, $t(6) = 5.98$, $P = 0.000979$. Data are presented as mean ± SEM, ***$P < 0.001$ compared to *Scramble*.

Source data are available online for this figure.

© 2021 The Authors

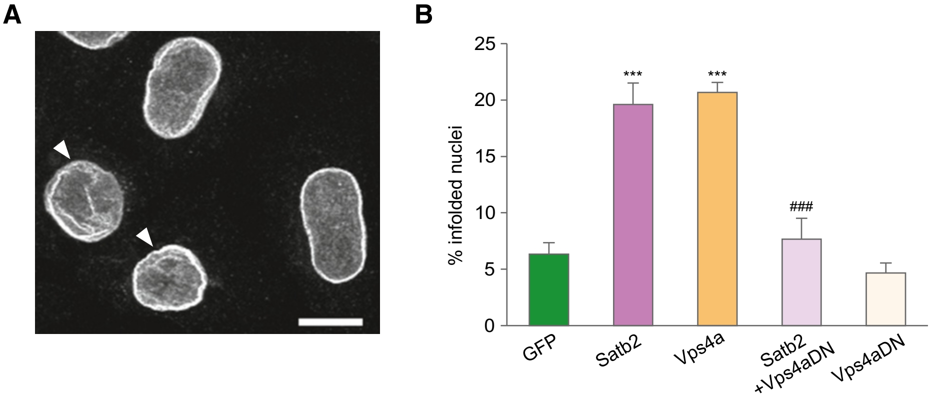

Figure EV3. 5 ESCRT-III/VPS4 complex is required for SATB2-triggered nuclear infolding formation in HeLa cells.

A Confocal image (*z*-axis projection of confocal image stack) of HeLa cell nuclei immunostained for Lamin B2. Arrowheads indicate infolded nuclei. Scale bar: 10 μm.

B Percentage of infolded nuclei in HeLa cells after ectopic expression of *Satb2*, *Vps4a-GFP*, a dominant-negative *Vps4a* mutant fused to GFP (*Vps4aDN-GFP*), and a combination of *Satb2* and *Vps4aDN-GFP*. The number of infolded nuclei is significantly increased in both *Satb2*- and *Vps4a*-transfected cells compared with GFP-transfected cells. Expression of the dominant-negative *Vps4a* mutant abolished the increase in the number of infolded nuclei induced by SATB2, $n = 3$–5 independent experiments, ANOVA followed by Tukey *post hoc* test, $F_{4,14} = 25.4$, *GFP* vs *Satb2*, $P < 0.0001$, *GFP* vs *Vps4a*, $P < 0.0001$, *Satb2* vs *Satb2* + *Vps4aDN*, $P = 0.0006$. Number of analyzed nuclei: 521 (*GFP*), 586 (*Satb2*), 302 (*Vps4a*), 345 (*Satb2*/*Vps4aDN*), 317 (*Vps4aDN*). Data are presented as mean ± SEM, ***$P < 0.0001$ compared to *GFP*; ###$P < 0.001$ compared to *Satb2*.

 © 2021 The Authors

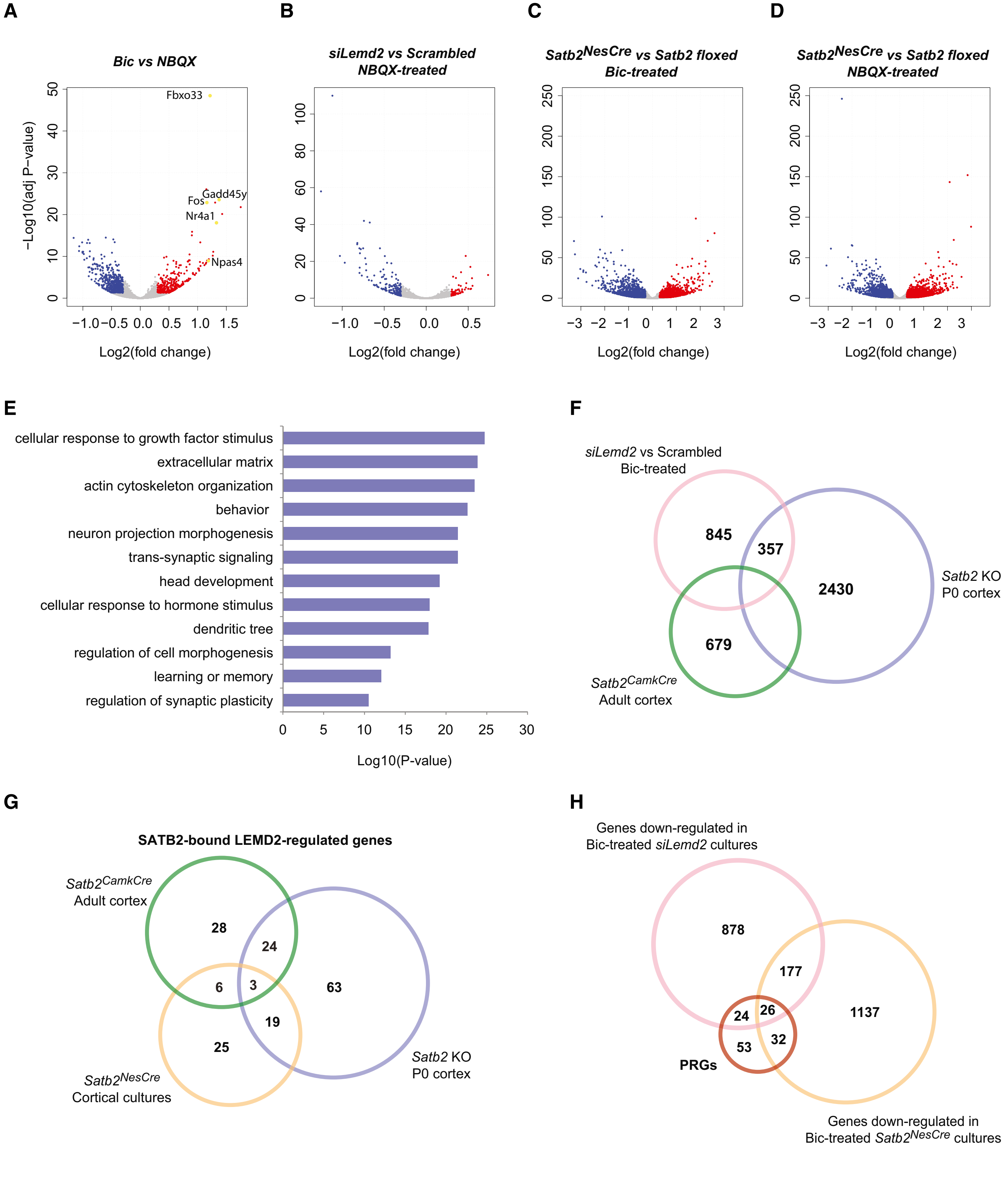
A
Bic vs NBQX
−Log10(adj P−value)
Fbxo33
Gadd45y
Fos
Nr4a1
Npas4
Log2(fold change)
B
siLemd2 vs Scrambled NBQX-treated
Log2(fold change)
C
Satb2NesCre vs Satb2 floxed Bic-treated
Log2(fold change)
D
Satb2NesCre vs Satb2 floxed NBQX-treated
Log2(fold change)
E
cellular response to growth factor stimulus
extracellular matrix
actin cytoskeleton organization
behavior
neuron projection morphogenesis
trans-synaptic signaling
head development
cellular response to hormone stimulus
dendritic tree
regulation of cell morphogenesis
learning or memory
regulation of synaptic plasticity
Log10(P-value)
F
siLemd2 vs Scrambled Bic-treated
845
357
2430
679
Satb2 KO P0 cortex
Satb2CamkCre Adult cortex
G
SATB2-bound LEMD2-regulated genes
Satb2CamkCre Adult cortex
28
24
6
3
63
19
25
Satb2NesCre Cortical cultures
Satb2 KO P0 cortex
H
Genes down-regulated in Bic-treated siLemd2 cultures
878
177
24
26
53
32
1137
PRGs
Genes down-regulated in Bic-treated Satb2NesCre cultures

Figure EV4.

Figure EV4. LEMD2 and SATB2 coregulate neuronal gene transcription.

A "Volcano plot" of statistical significance against fold change (FC) between silenced (NBQX-treated) and active (Bic-treated) primary cortical cultures from *Satb2* floxed mice. The differentially expressed genes (adjusted *P*-value < 0.05, 0.3 FC cut-off) are indicated in red (up-regulated) and in blue (down-regulated), n = 5–7 independent primary cultures. Examples of some IEGs strongly up-regulated upon Bic treatment are marked and highlighted in yellow.

B–D "Volcano plots" illustrating the differential gene expression between NBQX-treated *siLemd2*- vs scrambled siRNA-transfected cortical cultures (B), Bic-treated $Satb2^{NesCre}$ vs Satb2 floxed cultures (C), and NBQX-treated $Satb2^{NesCre}$ vs Satb2 floxed cultures (D). The differentially expressed genes (adjusted *P*-value < 0.05, 0.3 FC cut-off) are indicated in red (up-regulated) and in blue (down-regulated), n = 3–7 independent primary cultures.

E GO enrichment analysis of differentially expressed genes between siLemd2- and scrambled siRNA-transfected Bic-treated cultures.

F Venn diagram illustrating the overlap between the differentially expressed genes (adjusted *P*-value < 0.05, 0.3 FC cut-off) in *siLemd2*- vs scrambled siRNA-transfected cultures, SATB2-deficient vs wild-type P0 cortices (Fischer's exact test, *P*-value < 1E-15, OR = 2.93), and $Satb2^{CamkCre}$ vs *Satb2* floxed adult cortices (Fischer's exact test, *P*-value < 1E-15, OR = 3.54).

G Venn diagram of SATB2-bound LEMD2-regulated genes specific to or shared by the differentially expressed genes in $Satb2^{NesCre}$ cortical neurons (Fischer's exact test, *P*-value < 1E-15, OR = 3.059), P0 cortex (Fischer's exact test, *P*-value < 1E-15, OR = 2.627), and adult cortex (Fischer's exact test, *P*-value < 1E-15, OR = 2.962).

H Venn diagram showing the overlap between rapid and delayed PRG (Tyssowski *et al*, 2018) and the genes down-regulated in Bic-stimulated $Satb2^{NesCre}$ CKO (Fischer's exact test, *P*-value < 1E-15, OR = 9.99) and *siLemd2*-silenced cultures (Fischer's exact test, *P*-value < 1E-15, OR = 10.17).

 © 2021 The Authors
