# Supplementary material for: SATB2‐LEMD2 interaction links nuclear shape plasticity to regulation of cognition‐related genes
Source: EMBO J. 2020 Dec 15;40(3):e103701. doi: 10.15252/embj.2019103701 (PMC7849313; doi:10.15252/embj.2019103701)
Supplement: Supplementary file 8 — Source Data for Expanded View [file EMBJ-40-e103701-s008.pdf]

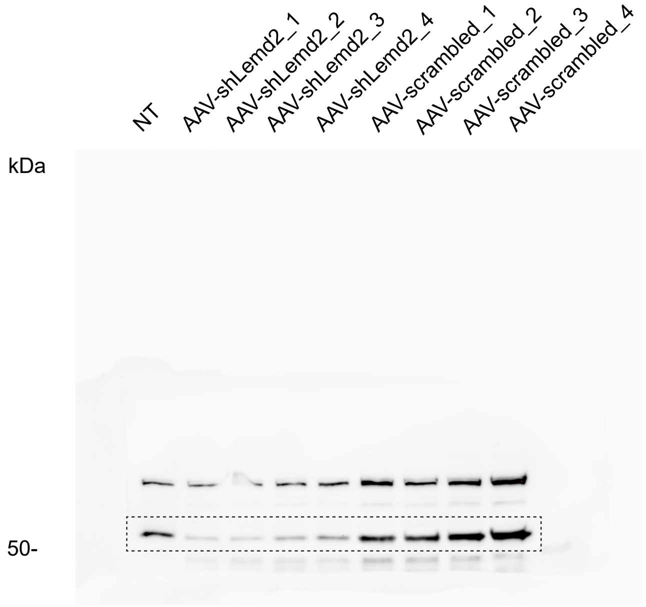

Cortical culture lysate
Detection: α-LEMD2

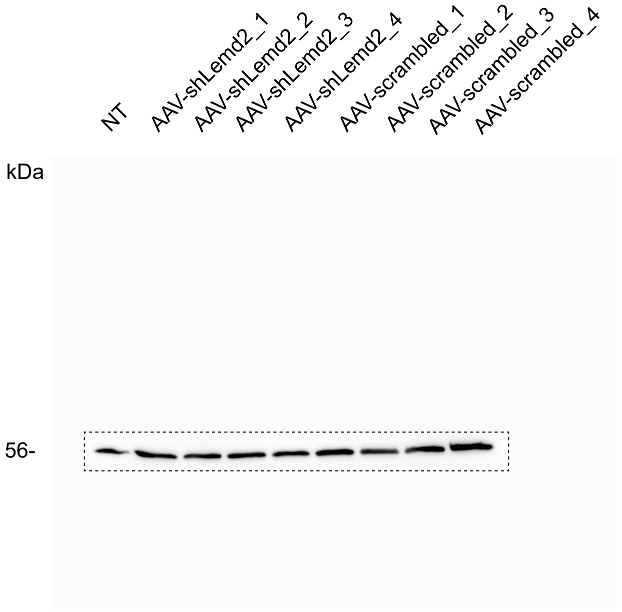

Cortical culture lysate
Detection: α-ßIII-Tubulin

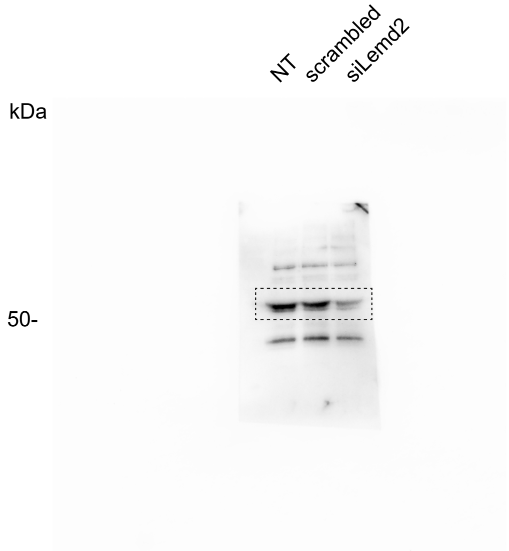

Hippocampal culture lysate
Detection: α-LEMD2

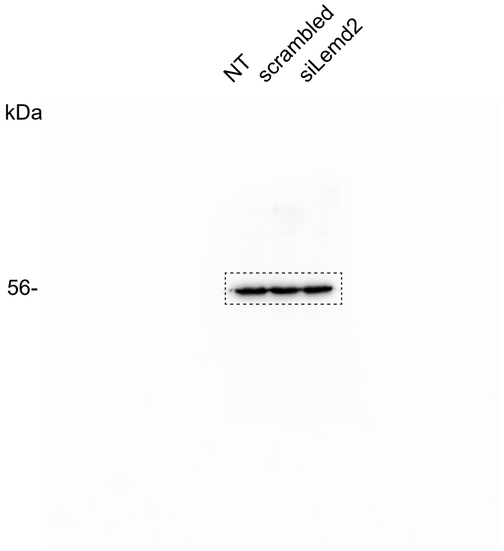

Hippocampal culture lysate
Detection: α-ßIII-Tubulin

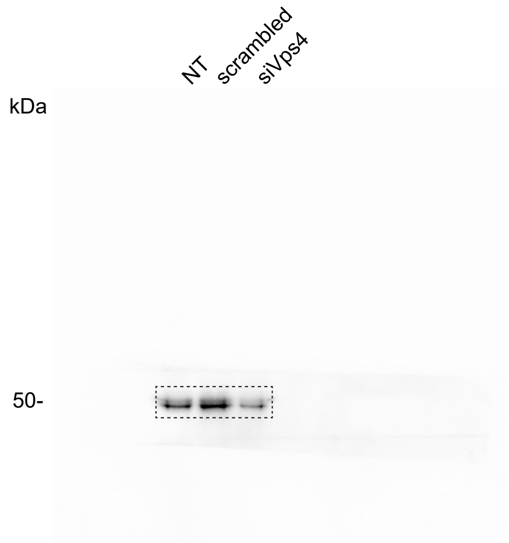

Hippocampal culture lysate
Detection: α-VPS4

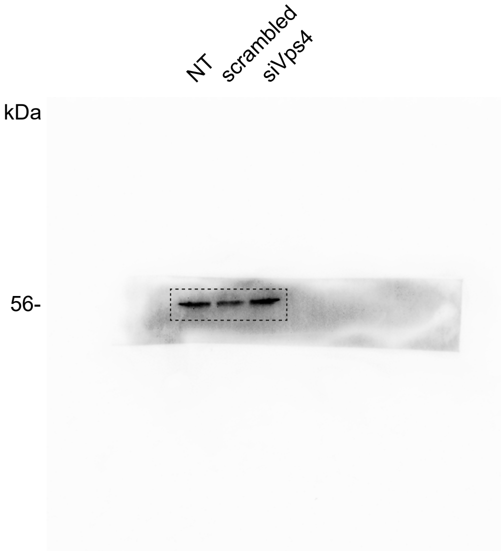

Hippocampal culture lysate
Detection: α-ßIII-Tubulin

Source Data EV Figure 2 | Uncropped Western blot membranes of silencing experiments

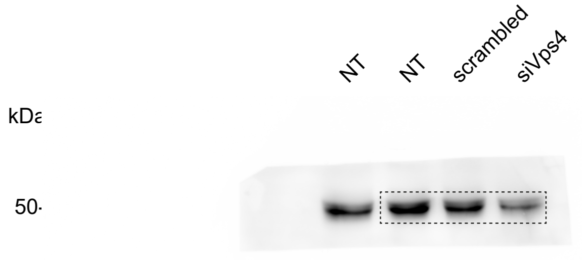

Cortical culture lysate
Detection: α-VPS4

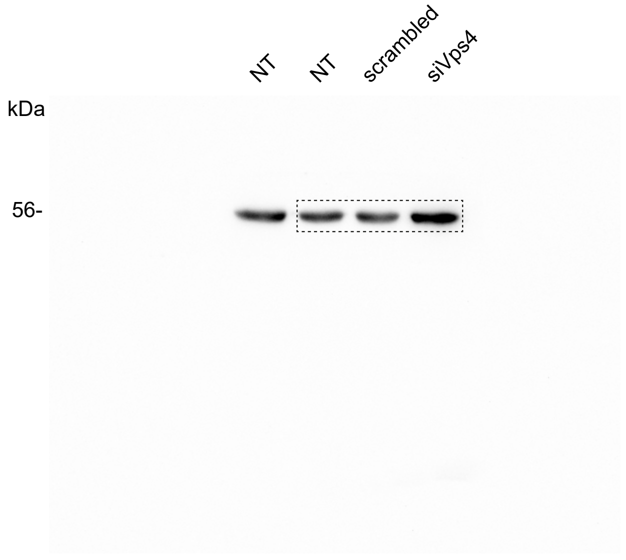

Cortical culture lysate
Detection: α-ßIII-Tubulin

Source Data EV Figure 2 | Uncropped Western blot membranes of silencing experiments
