# Supplementary material for: SATB2‐LEMD2 interaction links nuclear shape plasticity to regulation of cognition‐related genes
Source: EMBO J. 2020 Dec 15;40(3):e103701. doi: 10.15252/embj.2019103701 (PMC7849313; doi:10.15252/embj.2019103701)
Supplement: Supplementary file 9 — Reviw Process File [file EMBJ-40-e103701-s013.pdf]

THE EMBO JOURNAL

SATB2-LEMD2 interaction links nuclear shape plasticity to regulation of cognition-related genes

Patrick Feurle, Andreas Abentung, Isabella Cera, Nico Wahl, Cornelia Ablinger, Michael Bucher, Eduard Stefan, Simon Sprenger, David Teis, André Fischer, Aodán Laighneach, Laura Whitton, Derek Morris, Galina Apostolova, and Georg Dechant
DOI: N/A

Corresponding authors: Galina Apostolova (galina.apostolova@i-med.ac.at) , Georg Dechant (georg.dechant@i-med.ac.at)

Review Timeline:

Submission Date:	11th Oct 19
Editorial Decision:	22nd Nov 19
Revision Received:	11th Sep 20
Editorial Decision:	8th Oct 20
Revision Received:	22nd Oct 20
Accepted:	6th Nov 20

Editor: Ieva Gailite

Transaction Report:

(Note: With the exception of the correction of typographical or spelling errors that could be a source of ambiguity, letters and reports are not edited. Depending on transfer agreements, referee reports obtained elsewhere may or may not be included in this compilation. Referee reports are anonymous unless the Referee chooses to sign their reports.)

1st Editorial Decision 22nd Nov 2019

Thank you for submitting your manuscript for consideration by the EMBO Journal. We have now received three referee reports on your manuscript, which are included below for your information.

As you will see from the comments, reviewers #1 and #3 express interest in the work and appreciate the presented connection between Satb2-mediated regulation of nuclear shape and neuronal plasticity. While reviewer #2 is more critical, they also raise several partially overlapping concerns that need to be addressed in the revised version. Based on the interest expressed by reviewers #1 and #3, I would like to invite you to submit a revised version of your manuscript. Please address the comments of reviewers #1 and #3 and the related comments from reviewer #2 (points 5 and 9). Comments by reviewer #2 do not have to be addressed in full: addressing their points 4, 6, 7 and 8 is not required. However, please consider addressing at least in part the points 1, 2 and 3 from reviewer #2 (especially the electrophysiology/IEG response regulation aspects). Please note that public database deposition of acquired datasets is mandatory upon acceptance of the manuscript (point 10 by reviewer #2). I should add that it is The EMBO Journal policy to allow only a single major round of revision and that it is therefore important to resolve the main concerns at this stage.

We generally allow three months as standard revision time. Please contact us in advance if you would need an additional extension. As a matter of policy, competing manuscripts published during this period will not negatively impact on our assessment of the conceptual advance presented by your study. However, please contact me as soon as possible upon publication of any related work in order to discuss how to proceed.

When preparing your letter of response to the referees' comments, please bear in mind that this will form part of the Review Process File, and will therefore be available online to the community. For more details on our Transparent Editorial Process, please visit our website: https://www.embopress.org/page/journal/14602075/authorguide#transparentprocess

Please feel free to contact me if you have any further questions regarding the revision. Thank you for the opportunity to consider your work for publication. I look forward to receiving your revised manuscript.

Referee #1:

This is an extremely innovative paper exploring the cooperative function/interaction of SATB2 chromatin organizer and LEMD2 inner nuclear membrane protein and a subset of ESCRT protein complexes as regulator of the shape of neuronal nuclei, in context of activity-regulated paradigms and with implications for neuronal gene expression.
The paper presents a huge amount of work and in general has been conducted state of the art as it pertains to the co-immunoprecipitation experiments, the microscopy, with the hypothesis stringently tested in cell culture and in vivo mouse mutant models.
The paper is innovative and will have significant impact as the regulation of the 3D shape of neuronal nuclei is heavily understudied , with very little knowledge on molecular mechansism s thus far.

My only comment/criticsm is that there is one peculiar piece of evidence missing in this paper, which is the spatial proximity of genes jointly targeted by the SATB2-LEMD2 proteins to the inner nuclear membrane. One would expect that their SATB2-LEMD2 sensitive genes will show a change in their proximity to the inner nuclear membrane. could this be tested for at least some of the genes.
At the very least, this should be better discussed in the paper, or even better, tested experimentally.

Referee #2:

The manuscript by Feurle and colleagues investigates the interaction between SATB2 and LEMD2 and their role regulating activity-dependent changes of nuclear morphology and transcription. The most novel and interesting part of this study discusses the impact of Satb2 elimination and overexpression on activity-dependent changes of nuclear morphology. However, unfortunately, the project cannot effectively relate these changes in morphology and the reported protein interactions with the mechanisms regulating activity-driven transcription, nor provide novel insight into neuronal plasticity or memory processes.

Main criticisms:

1. At least three different studies have investigated the consequences of Satb2 elimination in neurons on mouse behavior and physiology. Two of these studies are referred in the text: Jaitner et al., 2016 and Li et al., 2017a. The third one by Zhang et al. 2019 is, however, not referred. The authors should discuss these results and refer whether the behavioral analyses were preformed in the same combination of floxed strain and cre driver. The article lacks a biological frame to interpret the relevance of the reported changes in nuclear morphology and gene expression. The authors should consider to include their own electrophysiological or behavioral analyses in the neuronal-specific KOs investigated here and strengthen the connection between Satb2, changes in nuclear morphology, and plasticity. For example, are the neurons responding to a given experience (i.e., Fos positive) more likely to present nuclear infoldings? Does this situation change in Stab2-cKOs in correlation with memory impairments?

2. After reporting that the elimination of Satb2 or Lemd2 impairs activity-dependent changes in nuclear morphology, it would be interesting to examine how these deficits affect activity-driven transcription. The decision of restricting the RNA-seq experiments to the stimulated condition, excluding the analysis of transcription in the basal condition, prevents a proper evaluation of gene induction. We do not know if the reported changes correspond to basal differences between genotypes or specifically emerge after stimulation. A 2x2 design (wt/cKO x Sal/Bic) would enable more analyses and might provide much more interesting results, particularly if the main objective is to relate the morphological changes with activity-dependent transcription and plasticity.

3. As summarized by the authors (p. 16), the study provides evidence that cooperation between SATB2 and other proteins of the nuclear envelope/lamina determine plastic changes in nuclear envelope geometry in response to action potential bursting. However, they did not conduct any experiment to explore the relevance and biological role of these changes. They later indicate (p. 18): "Consistent with this hypothesis, we did observe an impaired IEG response upon both SATB2 knockout and LEMD2 depletion in bicuculline-stimulated primary cortical neurons". Unfortunately, this sentence is not accurate the authors did not explore the IEG response, because to do that they would need to compare basal and induced levels in WT and cKO. As indicated above, this is one of the main caveats of the study.

4. The authors should demonstrate the specificity and efficiency of their conditional knockout. What is the percentage of neurons presenting Satb2 ablation? If the percentage is not close to 100% (as it happens in some CamK2a-cre lines), the authors could explore in the same slide the differential response and morphology of neurons expressing or lacking Satb2.

5. To complement the experiments in neuronal cultures, the authors should examine if activity-dependent changes of nuclear morphology are also impaired in vivo. For example, inducing status epilepticus with kainic acid or pilocarpine. It would be also very interesting to see if IEG induction (e.g., Fos) is affected in Satb2-KO cells and if the deficit somehow correlates with the impaired change in the nuclear envelope.

6. The experiment using rAAV to overexpress Satb2 could suggest that Satb2 is produced in response to response to activity and the activity-dependent increase in Satb2 levels causes the nuclear morphology change. This view is consistent with the results presented by Li and colleagues (2017). Is Satb2 also upregulated in the stimulation paradigms examined in this study?

7. In the same experiment, as they previously claimed that the infolding is triggered by the action potential, it would be interesting to block the action potential and examine if the overexpression still causes an increase in infolded nuclei.

8. The authors refer in the Discussion to a possible role of SATB2 and LEMD2 in the regulation of epigenetic marks (in particular they mention H3K9me2 ans H3K9me3). Given the current shortcomings of the study, it would be interesting that the authors strengthen their study by directly investigating the proposed connection between these proteins and the histone modifications.

9. It would be interesting to examine the spatial relocation in the nucleus (center to periphery) of some of the candidate genes identified in the transcriptome screen by fluorescence in situ hybridization (FISH) analysis. This would definitively strengthen the proposed model.

10. Availability of the datasets. The authors should indicate that their RNA-seq datasets have been deposited in a public database such as GEO and provide the access number in the manuscript. In addition, they should provide a token for access during peer review to the editor and reviewers. In most journals dealing with genomic data, the deposition of the datasets in a public repository is a mandatory requirement for publication.

Other comments:
11. The Abstract should be re-written to summarize better the objectives, results and conclusions of the study. It is surprising, for example, that the third sentence already states the main conclusion, when the results of the study have not been presented.

12. The results of the IPs and western-blot presented in Figure 1 should be quantified. This is advisable in all cases, but particularly important in the case of Figure 1A(iii) given the very subtle difference between co-IP SATB2 using the LEMD2 IgG antibodies. How do the authors explain the poor recovery compared to the other experiments.

13. In page 6, the authors study BAF, a protein that binds LEM domains. They prove that BAF is not important in the interaction between LEMD2 and SATB2. They should provide some background information about why they thought that BAF could be important in the interaction.

14. In page 6, line 17, the author mention that HeLa cells devoid of endogenous SATB2. They should probably mention this earlier because they also used HeLa in the GST experiment.

15. In Fig. 1C ii. Authors indicate SATB2(1-156) while in page 6 they refer to SATB2(1-157). Please, indicate which of them is correct.

16. Also, in page 7, line 4 the authors refer to "HD domain(346-733)"; but based on Figure 1 they likely meant to say (616-733).

17. P. 9: The authors wrote: "Injection with rAAV8-EGFP did not rescue the loss of infoldings in

Satb2-deficient CA1 pyramidal neurons (Fig 2E)". However, Figure 2E does not present this result and, supposedly, correspond to non-infected mice.

18. In page 10 line 9 and 10, the authors mention "a cocktail of three siRNAs targeting distinct parts of the Lemd2 mRNA 3'UTR reproducibly caused a strong reduction of LEMD2 (Fig 3A, I and Suppl. Fig 4A)". However it is not clear if the results presented in the Figures correspond to the cocktail or to an individual siRNA. This should be indicated in the legends. Also, the sequence of the siRNAs targeting Lemd2 mRNA should be provided.

19. In page 11, the authors claim that the "ectopic expression of STAB2 or VPS4 increased the number of infolded nuclei", as well as a dominant negative of Vps4 abolished the increase in infolded nuclei. However in Suppl Fig5 there is not image presenting these differences just a bar plot. Also the meaning of VPS4aDN is not explained (although I suppose it corresponds to the dominant negative mutant).

20. Fig. 4A and Suppl. Fig 7: Why did the authors decide to collapse the values higher than 6 in the Y-axis. These highly significant strongly up- and down-regulated genes re likely the most interesting candidates for further exploration. Are the IEGs located in this area?

21. The authors should probably highlight the axes and their intersection in the heatmap presented in Fig. 4C. The description of the graph is quite obscure, but it seems that a significant number of genes upregulated in cKOs show the opposite behavior in the siLemd2 experiment.

22. In page 13, line 14: Fig 3D does not exist, probably they refer to 4D.

23. In page 13, line 20: they said "62 genes were co-regulated by SATB2 in adult cortex", based in the numbers of Figure 4E, ii, there are 61 genes.

24. In Figure 4E, iii some negative genes should be added to illustrate the differences.

25. Some genome browser images showing representative genes differentially expressed in the RNA-seq analysis could be added to Fig. 4. It would be particularly interesting to present this together with the profile for Satb2 activity-dependent binding.

26. The first sentences of page 14 mention early and late response genes but do not cite the source of these lists and classification.

27. Also in page 14, line 7: the statistic needs a parenthesis to be reported in the same format than below.

28. In page 16, the authors claim that their analysis "reveals a previously unexpected overlap of the human phenotypes that converge on SATB2- and LEMD2-controlled gene-sets". They should test this by comparing the enrichments in the geneset corresponding to the overlap of the SATB2-KO and LEMD2-depleted screens and those exclusive of the SATB2 and LEMD2 screens.

Referee #3:

In the submitted manuscript, Feurle et al. describe the Satb2-Lemd2 interactions and their potential

role in the nuclear organization and gene expression regulation. Satb2 is an important chromatin regulator and transcription factor involved in the control of multiple aspects of brain development and functions. The authors have recently identified LEMD2 protein as and interacting partner of SATB2. LEMD2 is a nuclear protein implicated in organization of chromatin and nuclear structure.
In the current manuscript the authors characterized Satb2-Lemd2 both physical and genetic interactions. They show that Satb2 and LEMD2 interact physically via Cut domain on Satb2 and this interaction does not depend on BAF protein. On the other hand they show that Satb2 can control neuronal activity induced nuclear shape changes. Most importantly, in a series of genetic experiments, they show that Satb2 mediated nuclear folding induction requires Lemd2 as well as its partner, ATPase VPS4.
Next the authors compared gene expression changes in Satb2 and Lemd2 depleted neurons after bicucullin-treated neurons. Bicucullin is a GABA blocker that causes high levels of excitation and used in vitro to model neuronal activity. In these experiments the authors detected a number of genes that are regulated by both genes. Finally, the authors carried out computational analysis of Lemd2 regulated genes and found that some of these genes were mutated in human autism, schizophrenia and ID patients
To summarize: this is an impressive work that clearly shows a novel role of Satb2 in organizing nuclear shape as well as identifies its interacting partner. Both genetic and biochemical experiments are well designed and conducted. However, there are several week points as outlined below.

Involvement of Lemd2 in shaping neuronal nucleus is shown only in vitro, while for Satb2, the authors used both in vitro and in vivo approaches.
I suggest similar in vivo experiment for Lemd2. The authors seem to have all the tools, to transfect cortical or hippocampal neurons in vivo, without necessity of making a null allele. It would be interesting to see if absence of Lemd2 will have a similar phenotype in vivo, and whether it is Satb2 dependent.

Second problem is that the authors use bicucullin treatment as a model for activity induced genes. This is OK to a certain extent, however as any in vitro model has its limitations. On the other hand it is a model of epileptiform activity. Since it is a treatment that can cause robust non physiological changes, it can cause a lot of gene expression changes too that could be artefacts of global network malfunction. I would recommend the authors to be careful in their interpretations and tone down their conclusions about activity induced genes.

Response to reviewers

Referee #1:

This is an extremely innovative paper exploring the cooperative function/interaction of SATB2 chromatin organizer and LEMD2 inner nuclear membrane protein and a subset of ESCRT protein complexes as regulator of the shape of neuronal nuclei, in context of activity-regulated paradigms and with implications for neuronal gene expression.

The paper presents a huge amount of work and in general has been conducted state of the art as it pertains to the co-immunoprecipitation experiments, the microscopy, with the hypothesis stringently tested in cell culture and in vivo mouse mutant models.

The paper is innovative and will have significant impact as the regulation of the 3D shape of neuronal nuclei is heavily understudied , with very little knowledge on molecular mechansism s thus far.

My only comment/criticsm is that there is one peculiar piece of evidence missing in this paper, which is the spatial proximity of genes jointly targeted by the SATB2-LEMD2 proteins to the inner nuclear membrane. One would expect that their SATB2-LEMD2 sensitive genes will show a change in their proximity to the inner nuclear membrane. could this be tested for at least some of the genes.

At the very least, this should be better discussed in the paper, or even better, tested experimentally.

Response:

First, we would like to thank the reviewer for referring to our manuscript as “extremely innovative”. This comment provides very strong motivation for us.

We agree with the referee that studying nuclear localization and mobility relative to the inner nuclear membrane of LEMD2/SATB2-regulated loci upon neuronal activation is important. In order to address this question, we first successfully established immunofluorescence / DNA FISH in cortical cultures for *cFos* and *Gadd45γ* using protocol and probes described previously by Crepaldi et al. (PLoS Genet. 2013; 9(8):e1003699). An example of our experimental results is included below as a Figure (shown is a 2D LSM section of a cortical neuron nucleus, LaminB2-staining in red and cFos FISH signal in green). We next attempted to establish useful parameters for distance measurements between FISH signals and nuclear lamina (detected as Lamin B2-positive regions) in 3D-reconstructed nuclei. We soon realized that this is not a meaningful approach with the available image analysis tools due to the enormously complex 3D geometry of infolded nuclei. In the figure below arrows show only two examples of possible different 2D distances to the nuclear envelope that can be measured in an infolded nucleus. Obviously it becomes really complicated in 3D. We are currently establishing algorithms for clustering 3D-reconstructed neuronal nuclei according to their morphology using UMAP. This should allow us to measure mobility of FISH signals relative to complex shapes of the nuclear envelope in clusters rather than individual nuclei. While these experiments are ongoing, we are currently unable to provide reliable data. Therefore, we refrain from showing results of our preliminary experiments. Instead, we followed the recommendation of the reviewer to explain the situation in the discussion part of our manuscript.

Figure for referees removed.

Referee #2:

The manuscript by Feurle and colleagues investigates the interaction between SATB2 and LEMD2 and their role regulating activity-dependent changes of nuclear morphology and transcription. The most novel and interesting part of this study discusses the impact of Satb2 elimination and overexpression on activity-dependent changes of nuclear morphology. However, unfortunately, the project cannot effectively relate these changes in morphology and the reported protein interactions with the mechanisms regulating activity-driven transcription, nor provide novel insight into neuronal plasticity or memory processes.

Main criticisms:

1. At least three different studies have investigated the consequences of Satb2 elimination in neurons on mouse behavior and physiology. Two of these studies are referred in the text: Jaitner et al., 2016 and Li et al., 2017a. The third one by Zhang et al. 2019 is, however, not referred. The authors should discuss these results and refer whether the behavioral analyses were preformed in the same combination of floxed strain and cre driver.

Response:

We are aware of the publication by Zhang et al. 2019, however we fail to see its relevance for our study. This work describes experiments with a *Satb2 Emx1-Cre* knockout line that survives into adulthood allowing behavioral testing. It is not entirely clear why these animals survive while an - in essence identical - transgenic *Satb2 Emx1-Cre* line described previously by Sue McConnell's laboratory is perinatal lethal (Leone et al. Proc Natl Acad Sci U S A. 2015 Sep 15;112(37):11702-7). In order to avoid any uncertainty, we used *Nestin-Cre* as deleter. No Satb2 immunoreactivity is detected in neurons of these *Satb2*^{NesCre} conditional knockout mice (see Cera et al. 2019 and Appendix Figure 1). Consistent with the report by Leone et al. (Cerebral Cortex (2015) 25(10) 3406-3419) for their *Emx1-Cre* driven *Satb2* knockout, our *Satb2*^{NesCre} conditional knockout animals lack corpus callosum and die for unknown reasons at around P30.

This precludes any behavioral analyses of these mutants. Instead, we use $Satb2^{CamkCre}$ conditional knockout mice for behavioral studies and analyses of adult brain. This line was described in detail in one of our previous publications (Jaitner et. al. 2016, Elife *5*, e17361). The reasoning behind using these 2 different *Satb2* cKO lines in our study is now outlined explicitly in the manuscript and we have added a supplementary figure describing the $\textit{Satb2}^{NesCre}$ phenotype (Appendix Figure 1).

The article lacks a biological frame to interpret the relevance of the reported changes in nuclear morphology and gene expression. The authors should consider to include their own electrophysiological or behavioral analyses in the neuronal-specific KOs investigated here and strengthen the connection between Satb2, changes in nuclear morphology, and plasticity. For example, are the neurons responding to a given experience (i.e., Fos positive) more likely to present nuclear infoldings? Does this situation change in Stab2-cKOs in correlation with memory impairments?

Response:

We have added the requested behavioral experiment in adult animals. Using $Satb2^{CamkCre}$ mice, for which we have previously described deficient L-LTP and long-term memory, allowed for the correlation of cFos expression, nuclear infolding and memory deficit. To study the correlation between nuclear infoldings and neuronal activation *in vivo*, we used exposure to novel environment as an experimental model. In contrast to pharmacologically-triggered neuronal activation, e.g. KA-induced status epilepticus that causes strong and synchronous activation in all hippocampal subfields, the exploration of a novel and rich spatial context is an established behavioral paradigm that activates sparse neuronal assemblies throughout the hippocampus by a natural stimulus, thus allowing for the analysis of both activated (cFos-positive) and inactive (cFos-negative) neurons. As outlined in the modified manuscript as well as in the novel Fig 5, the results show that the deep invaginations of the nuclear membrane are a correlate of neuronal activation triggered by a naturalistic stimulus and that SATB2 is required for both nuclear envelope plasticity and cFos induction *in vivo*.

2. After reporting that the elimination of Satb2 or Lemd2 impairs activity-dependent changes in nuclear morphology, it would be interesting to examine how these deficits affect activity-driven transcription. The decision of restricting the RNA-seq experiments to the stimulated condition, excluding the analysis of transcription in the basal condition, prevents a proper evaluation of gene induction. We do not know if the reported changes correspond to basal differences between genotypes or specifically emerge after stimulation. A 2x2 design (wt/cKO x Sal/Bic) would enable more analyses and might provide much more interesting results, particularly if the main objective is to relate the morphological changes with activity-dependent transcription and plasticity.

Response:

New sequencing data have been added to the manuscript. We first tested validity of our neuronal *in vitro* stimulation paradigm by transcriptome sequencing of control Satb2 floxed neurons under stimulated (Bic-treated) and moderately active/inhibited (NBQX-treated) conditions. We found the expected regulation of activity-dependent genes described in two previous papers: Tyssowski, K.M.et al. (Neuron *98*, 530-546.e11, 2018) and Hrvatin et al. (Nat. Neurosci. *21*, 120–129, 2018) as references (Figure EV4). We than tested the effect of both *Lemd2* and *Satb2* loss-of-function in both the stimulated and inhibited condition in the requested 2x2 design. The results of these experiments on the global transcriptome are depicted in the new Fig 4 and the new Fig EV4. The effect of SATB2 and LEMD2 deficiency on activity-regulated genes under both conditions is also depicted in new panels in Fig 4 and Fig EV4 and Table EV3. The new results confirm and expand our previous results of robustly overlapping gene sets under all conditions for the global transcriptome and in particular for activity-dependent genes.

3. As summarized by the authors (p. 16), the study provides evidence that cooperation between SATB2 and other proteins of the nuclear envelope/lamina determine plastic changes in nuclear envelope geometry in response to action potential bursting. However, they did not conduct any experiment to explore the relevance and biological role of these changes. They

later indicate (p. 18): "Consistent with this hypothesis, we did observe an impaired IEG response upon both SATB2 knockout and LEMD2 depletion in bicuculline-stimulated primary cortical neurons". Unfortunately, this sentence is not accurate the authors did not explore the IEG response, because to do that they would need to compare basal and induced levels in WT and cKO. As indicated above, this is one of the main caveats of the study.

Response:

As outlined above, we have added a comparison of transcriptomes obtained under stimulated and inhibited conditions in SATB2-deficient and *Lemd2*-knockdown cultures. In addition, we addressed the biological relevance of *Satb2* for the stimulation response by analysis of cFos as an established marker of neuronal activiation in hippocampal neurons *in vivo* after exposure of freely behaving adult mice to stimulation by novel environment.

4. The authors should demonstrate the specificity and efficiency of their conditional knockout. What is the percentage of neurons presenting Satb2 ablation? If the percentage is not close to 100% (as it happens in some CamK2a-cre lines), the authors could explore in the same slide the differential response and morphology of neurons expressing or lacking Satb2.

Response:

Both conditional *Satb2* knockout lines employed in this study have non-detectable levels of SATB2 in all neurons (Nestin-Cre) or adult pyramidal forebrain neurons (CamkII-Cre). For the CamkII-Cre line this is documented in Jaitner et al, eLIFE 2016. For the Nestin-Cre line, which we first described in Cera et.al 2019 PLOS Genet. *15*, e1007890, we have provided additional evidence in Appendix Fig 1.

We are very grateful for the suggestion to analyze a mosaic of *Satb2*-deficient and *Satb2*-expressing cells in the same histological sections. This was not possible for *Satb2* due to the complete nature of the transgenic modification as stated above. However, motivated by the reviewer's comment, we followed such a mosaic-analysis strategy for our *in vivo Lemd2* knockdown analysis as will be outlined below.

5. To complement the experiments in neuronal cultures, the authors should examine if activity-dependent changes of nuclear morphology are also impaired in vivo. For example, inducing status epilepticus with kainic acid or pilocarpine. It would be also very interesting to see if IEG induction (e.g., Fos) is affected in Satb2-KO cells and if the deficit somehow correlates with the impaired change in the nuclear envelope.

Response:

Here we refer to our response given to point number 1. The requested experiment has been added, however instead of using harsh pharmacological stimulation, we decided to stimulate neurons with natural sensory stimulation by exposure to novel environment.

6. The experiment using rAAV to overexpress Satb2 could suggest that Satb2 is produced in response to response to activity and the activity-dependent increase in Satb2 levels causes the nuclear morphology change. This view is consistent with the results presented by Li and colleagues (2017). Is Satb2 also upregulated in the stimulation paradigms examined in this study?

Response:

We have characterized the effect of neuronal activation and BDNF on SATB2 levels in hippocampal cultures in substantial detail. This work, we would like to point out, was already published in 2016 (Jaitner et al. eLIFE 2016). We found that while both neuronal activity and BDNF stimulated *Satb2* expression, the kinetics of both activity-dependent *Satb2* up-regulation after stimulation as well as *Satb2* down-regulation after inhibition with Trk inhibitor K252a occur with a half-life of 6 – 12 h. By contrast, nuclear infolding occurs with fast kinetics within an hour (Wittmann et al. 2009). Therefore, activity-dependent regulation of SATB2 is unlikely to explain *Satb2*-dependent infolding of the nuclear envelope in stimulated neurons. It appears more likely that SATB2 undergoes activity-dependent homo di- or tetramerisation or post-translational modification. However, this remains to be established in future experiments.

7. In the same experiment, as they previously claimed that the infolding is triggered by the action potential, it would be interesting to block the action potential and examine if the overexpression still causes an increase in infolded nuclei.

Response:

We have tested the effect of overexpression of *Satb2* on infoldings in hippocampal neurons in the absence of Bic-stimulation. Since under these conditions neurons are only sparsely active, further pharmacological inhibition will have no or very little effect.

8. The authors refer in the Discussion to a possible role of SATB2 and LEMD2 in the regulation of epigenetic marks (in particular they mention H3K9me2 ans H3K9me3). Given the current shortcomings of the study, it would be interesting that the authors strengthen their study by directly investigating the proposed connection between these proteins and the histone modifications.

Response:

We agree with the referee that analyses of *Satb2*-dependent alterations in epigenetic marks are important. However, we fail to see an immediate link to the content of the present study focusing on nuclear shape. These epigenetic analyses are part of a separate study, in which we will describe the effect of *Satb2* on 3D chromatin configuration in pyramidal neurons.

9. It would be interesting to examine the spatial relocation in the nucleus (center to periphery) of some of the candidate genes identified in the transcriptome screen by fluorescence in situ hybridization (FISH) analysis. This would definitively strengthen the proposed model.

Response:

We refer to our response given to the same point raised by Reviewer #1

10. Availability of the datasets. The authors should indicate that their RNA-seq datasets have been deposited in a public database such as GEO and provide the access number in the manuscript. In addition, they should provide a token for access during peer review to the editor and reviewers. In most journals dealing with genomic data, the deposition of the datasets in a public repository is a mandatory requirement for publication.

Response:

All transcriptome data sets have been deposited as requested by the policy of EMBO J.

Other comments:

11. The Abstract should be re-written to summarize better the objectives, results and conclusions of the study. It is surprising, for example, that the third sentence already states the main conclusion, when the results of the study have not been presented.

Response:

The abstract has been modified according to this comment.

12. The results of the IPs and western-blot presented in Figure 1 should be quantified. This is advisable in all cases, but particularly important in the case of Figure 1A(iii) given the very subtle difference between co-IP SATB2 using the LEMD2 IgG antibodies. How do the authors explain the poor recovery compared to the other experiments.

Response:

Western blot raw data are provided following EMBO J guidelines. Two different explanations for the comparably weak LEMD2 signal can be put forward. First, the available antibodies for LEMD2 are of poor quality in stark contrast to SATB2 antibodies. Second, it appears likely that only a minor fraction of LEMD2 is bound to SATB2 in primary neurons and can be co-

immunoprecipitated with SATB2. Due to these limitations, we deemed it important to corroborate the protein interaction between SATB2 and LEMD2 with recombinant tagged versions of both proteins. As is evident from the results depicted in Fig.1, these experiments provided evidence for very robust and strong interactions between SATB2 and LEMD2.

13. In page 6, the authors study BAF, a protein that binds LEM domains. They prove that BAF is not important in the interaction between LEMD2 and SATB2. They should provide some background information about why they thought that BAF could be important in the interaction.

Response:

The manuscript has been modified accordingly.

14. In page 6, line 17, the author mention that HeLa cells devoid of endogenous SATB2. They should probably mention this earlier because they also used HeLa in the GST experiment.

Response:

The manuscript has been modified accordingly.

15. In Fig. 1C ii. Authors indicate SATB2(1-156) while in page 6 they refer to SATB2(1-157). Please, indicate which of them is correct.

Response:

The reviewer is correct and we apologize for the mistake. The manuscript has been corrected.

16. Also, in page 7, line 4 the authors refer to "HD domain(346-733)"; but based on Figure 1 they likely meant to say (616-733).

Response:

Again the reviewer is correct. The manuscript has been corrected.

17. P. 9: The authors wrote: "Injection with rAAV8-EGFP did not rescue the loss of infoldings in Satb2-deficient CA1 pyramidal neurons (Fig 2E)". However, Figure 2E does not present this result and, supposedly, correspond to non-infected mice.

Response:

Figure 2 has been reorganized in the revised manuscript.

18. In page 10 line 9 and 10, the authors mention "a cocktail of three siRNAs targeting distinct parts of the Lemd2 mRNA 3'UTR reproducibly caused a strong reduction of LEMD2 (Fig 3A, I and Suppl. Fig 4A)". However it is not clear if the results presented in the Figures correspond to the cocktail or to an individual siRNA. This should be indicated in the legends. Also, the sequence of the siRNAs targeting Lemd2 mRNA should be provided.

Response:

We have used commercially available cocktails of siRNAs (targeting *Lemd2, Vps4a, Vps4b* and scrambled siRNA) with proprietary sequences not disclosed to the customer. The order numbers of the cocktails are provided in the manuscript. For the revision of the manuscript we have generated AAV-shRNA viruses which we then used to knockdown Lemd2 *in vitro* and *in vivo*. We have designed these shRNAs and the sequences are described in the revised manuscript. We found results to be consistent between siRNA and shRNA knockdown in all comparisons.

19. In page 11, the authors claim that the "ectopic expression of STAB2 or VPS4 increased the number of infolded nuclei", as well as a dominant negative of Vps4 abolished the increase in infolded nuclei. However in Suppl Fig5 there is not image presenting these differences just a bar plot. Also the meaning of VPS4aDN is not explained (although I suppose it corresponds to the dominant negative mutant).

Response:

We decided against showing these infoldings because their appearance is not principally different from the other infolded nuclei depicted in the manuscript rendering their display redundant information. The reviewer is correct, DN stands for dominant negative. This information has been added to the manuscript.

20. Fig. 4A and Suppl. Fig 7: Why did the authors decide to collapse the values higher than 6 in the Y-axis. These highly significant strongly up- and down-regulated genes re likely the most interesting candidates for further exploration. Are the IEGs located in this area?

Response:

The entire figure 4 has been reorganized. New data have been added in the format of Volcano blots. The effect on IEGs specifically is described in Table EV3.

21. The authors should probably highlight the axes and their intersection in the heatmap presented in Fig. 4C. The description of the graph is quite obscure, but it seems that a significant number of genes upregulated in cKOs show the opposite behavior in the siLemd2 experiment.

Response:

The reviewer is correct. Some genes are regulated in opposite direction by *Satb2* versus *Lemd2* loss-of-function. All relevant information about individual genes is available in the deposited GEO dataset. IEG expression in general is affected in the same direction for individual genes, up- or down-regulated, by *Satb2* or *Lemd2* loss-of-function.

22. In page 13, line 14: Fig 3D does not exist, probably they refer to 4D.

Response:

The manuscript has been corrected.

23. In page 13, line 20: they said "62 genes were co-regulated by SATB2 in adult cortex", based in the numbers of Figure 4E, ii, there are 61 genes.

Response:

These figures have changed (increased) due to the addition of more sequencing information.

24. In Figure 4E, iii some negative genes should be added to illustrate the differences.

Response:

We are not sure what the referee is referring to with "negative genes".

25. Some genome browser images showing representative genes differentially expressed in the RNA-seq analysis could be added to Fig. 4. It would be particularly interesting to present this together with the profile for Satb2 activity-dependent binding.

Response:

Figure 4 panel G depicts a combination of genome browser images of *Lemd2* regulated genes combined with ChIP-seq data for SATB2.

26. The first sentences of page 14 mention early and late response genes but do not cite the source of these lists and classification.

Response:

Our reference lists for activity-dependent genes stem from Tyssowski, K.M.et al. (2018) Neuron *98*, 530-546.e11. and Hrvatin et al., (2018) Nat. Neurosci. *21*, 120–129. Both papers were already cited in the previous version of the manuscript. In the revised version they are now explicitedly referenced in the Results part.

27. Also in page 14, line 7: the statistic needs a parenthesis to be reported in the same format than below.

Response:

The Manuscript has been modified accordingly.

28. In page 16, the authors claim that their analysis "reveals a previously unexpected overlap of the human phenotypes that converge on SATB2- and LEMD2-controlled gene-sets". They should test this by comparing the enrichments in the geneset corresponding to the overlap of the SATB2-KO and LEMD2-depleted screens and those exclusive of the SATB2 and LEMD2 screens.

Response:

The primary motivation for the GSA analyses was to characterize the relevance of the novel LEMD2-regulated gene set for human phenotypes. We were surprised to observe that it is associated with in essence the same phenotypes that we have reported before for SATB2-dependent genes. Restricting the analysis to the commonly regulated gene sets would drastically reduce statistical power and consequently the quality of the results.

Referee #3:

In the submitted manuscript, Feurle et al. describe the Satb2-Lemd2 interactions and their potential role in the nuclear organization and gene expression regulation. Satb2 is an important chromatin regulator and transcription factor involved in the control of multiple aspects of brain development and functions. The authors have recently identified LEMD2 protein as and interacting partner of SATB2. LEMD2 is a nuclear protein implicated in organization of chromatin and nuclear structure.

In the current manuscript the authors characterized Satb2-Lemd2 both physical and genetic interactions. They show that Satb2 and LEMD2 interact physically via Cut domain on Satb2 and this interaction does not depend on BAF protein. On the other hand they show that Satb2 can control neuronal activity induced nuclear shape changes. Most importantly, in a series of genetic experiments, they show that Satb2 mediated nuclear folding induction requires Lemd2 as well as its partner, ATPase VPS4.

Next the authors compared gene expression changes in Satb2 and Lemd2 depleted neurons after bicucullin-treated neurons. Bicucullin is a GABA blocker that causes high levels of excitation and used in vitro to model neuronal activity. In these experiments the authors detected a number of genes that are regulated by both genes. Finally, the authors carried out computational analysis of Lemd2 regulated genes and found that some of these genes were mutated in human autism, schizophrenia and ID patients

To summarize: this is an impressive work that clearly shows a novel role of Satb2 in organizing nuclear shape as well as identifies its interacting partner. Both genetic and biochemical experiments are well designed and conducted. However, there are several week points as outlined below.

Involvement of Lemd2 in shaping neuronal nucleus is shown only in vitro, while for Satb2, the authors used both in vitro and in vivo approaches.

I suggest similar in vivo experiment for Lemd2. The authors seem to have all the tools, to transfect cortical or hippocampal neurons in vivo, without necessity of making a null allele. It would be interesting to see if absence of Lemd2 will have a similar phenotype in vivo, and whether it is Satb2 dependent.

Response:

We agree with the referee that studying the effect of LEMD2 depletion on nuclear morphology is important. Unfortunately, siRNA-mediated knockdown, while very useful *in vitro*, is practically impossible *in vivo* due to the unstable nature of the agent. We are not aware of relevant publications applying this method in CNS. Hence, in order to perform the requested experiment, we generated and characterized shRNA expressing AAV viruses which turned out to very effectively reduce LEMD2 protein levels. As additional advantage, these viruses also encode mCherry as fluorescent marker allowing us to discriminate virus-transduced from non-transduced neurons on identical sections. We are happy to report in the revised manuscript that upon stereotactic injection of these viruses into adult hippocampus our analyses of mosaic sections containing both tranduced and non-transduced neurons yielded highly significant results demonstrating that *Lemd2* knockdown in fact causes deficit in nuclear infolding of pyramidal neurons *in vivo* .

Second problem is that the authors use bicucullin treatment as a model for activity induced genes. This is OK to a certain extent, however as any in vitro model has its limitations. On the other hand it is a model of epileptiform activity. Since it is a treatment that can cause robust non physiological changes, it can cause a lot of gene expression changes too that could be artefacts of global network malfunction. I would recommend the authors to be careful in their interpretations and tone down their conclusions about activity induced genes.

Response:

Again, the reviewer is making a very valid point. Bic treatment has been used in a large number of studies to force neuronal primary cultures into bouts of activity. The level of activation by Bic

remains close to what can be described as physiological levels of activity for example as compared to the classic method of depolarization by KCl. However, we agree that pharmacological treatment is not a good option *in vivo*. Therefore, we chose the naturalistic sensory stimulation of novel environmental stimulation for our *in vivo* analyses that were conducted for the revision of our manuscript. The result of this *in vivo* stimulation paradigm is described in the new Figure 5. It is consistent with what we observed in Bic-treated cultures *in vitro*. In addition, we have modified the manuscript following the reviewer's advice.

Thank you for submitting a revised version of your manuscript. Your study has now been seen by two of the original referees, who find that most of their main concerns have been addressed and support publication of the revised manuscript. There now remain only a few editorial issues that have to be addressed before I can extend formal acceptance of the manuscript.

Referee #2:

The article presents a number of new experiments and has been greatly improved. I find the novelty exposure experiment presented in the new Fig. 5 particularly compelling and important to support authors' conclusions. The extended transcriptome analysis also strengthened the study. I therefore support the acceptance of the manuscript.

Referee #3:

In the revised version, the authors addressed my concerns about Lemd KD in vivo experiments as well as bicuculline treatment. They also performed experiments suggested by other reviewers.

2nd Authors' Response to Reviewers 22nd Oct 2020

The authors performed the requested changes.

2nd Revision - Editorial Decision 6th Nov 2020

Editor accepted the manuscript.

EMBO PRESS

YOU MUST COMPLETE ALL CELLS WITH A PINK BACKGROUND ➔

PLEASE NOTE THAT THIS CHECKLIST WILL BE PUBLISHED ALONGSIDE YOUR PAPER

Corresponding Author Name: Galina Apostolova
Journal Submitted to: EMBO Journal
Manuscript Number: EMBOJ-2019-103701

Reporting Checklist For Life Sciences Articles (Rev. June 2017)

This checklist is used to ensure good reporting standards and to improve the reproducibility of published results. These guidelines are consistent with the Principles and Guidelines for Reporting Preclinical Research issued by the NIH in 2014. Please follow the journal's authorship guidelines in preparing your manuscript.

A- Figures

1. Data

The data shown in figures should satisfy the following conditions:

- ➔ the data were obtained and processed according to the field's best practice and are presented to reflect the results of the experiments in an accurate and unbiased manner.
- ➔ figure panels include only data points, measurements or observations that can be compared to each other in a scientifically meaningful way.
- ➔ graphs include clearly labeled error bars for independent experiments and sample sizes. Unless justified, error bars should not be shown for technical replicates.
- ➔ if n< 5, the individual data points from each experiment should be plotted and any statistical test employed should be justified
- ➔ Source Data should be included to report the data underlying graphs. Please follow the guidelines set out in the author ship guidelines on Data Presentation.

2. Captions

Each figure caption should contain the following information, for each panel where they are relevant:

- ➔ a specification of the experimental system investigated (eg cell line, species name).
- ➔ the assay(s) and method(s) used to carry out the reported observations and measurements
- ➔ an explicit mention of the biological and chemical entity(ies) that are being measured.
- ➔ an explicit mention of the biological and chemical entity(ies) that are altered/varied/perturbed in a controlled manner.

- ➔ the exact sample size (n) for each experimental group/condition, given as a number, not a range;
- ➔ a description of the sample collection allowing the reader to understand whether the samples represent technical or biological replicates (including how many animals, litters, cultures, etc.).
- ➔ a statement of how many times the experiment shown was independently replicated in the laboratory.
- ➔ definitions of statistical methods and measures:
  - common tests, such as t-test (please specify whether paired vs. unpaired), simple χ^2 tests, Wilcoxon and Mann-Whitney tests, can be unambiguously identified by name only, but more complex techniques should be described in the methods section;
  - are tests one-sided or two-sided?
  - are there adjustments for multiple comparisons?
  - exact statistical test results, e.g., P values = x but not P values < x;
  - definition of 'center values' as median or average;
  - definition of error bars as s.d. or s.e.m.

Any descriptions too long for the figure legend should be included in the methods section and/or with the source data.

In the pink boxes below, please ensure that the answers to the following questions are reported in the manuscript itself. Every question should be answered. If the question is not relevant to your research, please write NA (non applicable). We encourage you to include a specific subsection in the methods section for statistics, reagents, animal models and human subjects.

USEFUL LINKS FOR COMPLETING THIS FORM

http://www.antibodypedia.com
http://1degreebio.org
http://www.equator-network.org/reporting-guidelines/improving-bioscience-research-repor
http://grants.nih.gov/grants/olaw/olaw.htm
http://www.mrc.ac.uk/Ourresearch/Ethicsresearchguidance/Useofanimals/index.htm
http://ClinicalTrials.gov
http://www.consort-statement.org
http://www.consort-statement.org/checklists/view/32-consort/66-title
http://www.equator-network.org/reporting-guidelines/reporting-recommendations-for-tumo
http://datadryad.org
http://figshare.com
http://www.ncbi.nlm.nih.gov/gap
http://www.ebi.ac.uk/ega
http://biomodels.net/
http://biomodels.net/miriam/
http://jjj.biochem.sun.ac.za
http://oba.od.nih.gov/biosecurity/biosecurity_documents.html
http://www.selectagents.gov/

B- Statistics and general methods

Please fill out these boxes ➔ (Do not worry if you cannot see all your text once you press return)

1.a. How was the sample size chosen to ensure adequate power to detect a pre-specified effect size?	Sample sizes varied to obtain interpretable results. They were not statistically pre-determined but were similar to sample sizes generally employed in the field.
1.b. For animal studies, include a statement about sample size estimate even if no statistical methods were used.	Sample sizes varied to obtain interpretable results. They were not statistically pre-determined but were similar to sample sizes generally employed in the field.
2. Describe inclusion/exclusion criteria if samples or animals were excluded from the analysis. Were the criteria pre-established?	No exclusion criteria for data points were pre-established.
3. Were any steps taken to minimize the effects of subjective bias when allocating animals/samples to treatment (e.g. randomization procedure)? If yes, please describe.	Sister primary cultures for all analyses were randomly assigned to the experimental groups. Animals were randomly taken from the home cage and assigned to treatment.
For animal studies, include a statement about randomization even if no randomization was used.	Randomization was used as described above.
4.a. Were any steps taken to minimize the effects of subjective bias during group allocation or/and when assessing results (e.g. blinding of the investigator)? If yes please describe.	For quantification of all experiments the investigator was blinded by assigning random nummerizations to the samples (e.g. Group A, B, C, etc.)
4.b. For animal studies, include a statement about blinding even if no blinding was done	Behavioral animal study was performed under blinded to the genotype conditions.
5. For every figure, are statistical tests justified as appropriate?	Statistical tests are justified as appropriate for each calculated comparison.
Do the data meet the assumptions of the tests (e.g., normal distribution)? Describe any methods used to assess it.	Appropriate statistical tests were employed. Normal distribution was tested by Shapiro-Wilk test.
Is there an estimate of variation within each group of data?	Equal variance between groups was tested by Levene's test.

Is the variance similar between the groups that are being statistically compared?	Yes

C- Reagents

6. To show that antibodies were profiled for use in the system under study (assay and species), provide a citation, catalog number and/or clone number, supplementary information or reference to an antibody validation profile. e.g., Antibodypedia (see link list at top right), 1DegreeBio (see link list at top right).	Commercial antibodies were used in the experiments. We relied on the validation data reported by the vendor. Antibodies have been validated for the specific applications. Antibody catalog numbers are listed in the "Methods" section.
7. Identify the source of cell lines and report if they were recently authenticated (e.g., by STR profiling) and tested for mycoplasma contamination.	HeLa cell line was obtained form ATCC cell bank. The cell line was tested for mycoplasma contamination.

* for all hyperlinks, please see the table at top right of the document

D- Animal Models

8. Report species, strain, gender, age of animals and genetic modification status where applicable. Please detail housing and husbandry conditions and the source of animals.	Mus Musculus, C57Bl6, males, Satb2flx/flx::Camk2a-Cre (C57Bl6 background), Satb2flx/flx::Nes-Cre (C57Bl6 background), Satb2flx/flx (C57Bl6 background), 3 months of age. Genetically modified animals were generated by our group. Wild-type mice (C57Bl6 strain) were obtained from Charles River. Mice were housed under standard housing conditions. Environmental enrichment like nesting material, mouse homes and gnawing sticks were provided.
9. For experiments involving live vertebrates, include a statement of compliance with ethical regulations and identify the committee(s) approving the experiments.	All experimental procedures were approved by the Austrian Animal Experimentation Ethics Board.
10. We recommend consulting the ARRIVE guidelines (see link list at top right) (PLoS Biol. 8(6), e1000412, 2010) to ensure that other relevant aspects of animal studies are adequately reported. See author guidelines, under 'Reporting Guidelines'. See also: NIH (see link list at top right) and MRC (see link list at top right) recommendations. Please confirm compliance.	Yes

E- Human Subjects

11. Identify the committee(s) approving the study protocol.	NA
12. Include a statement confirming that informed consent was obtained from all subjects and that the experiments conformed to the principles set out in the WMA Declaration of Helsinki and the Department of Health and Human Services Belmont Report.	NA
13. For publication of patient photos, include a statement confirming that consent to publish was obtained.	NA
14. Report any restrictions on the availability (and/or on the use) of human data or samples.	NA
15. Report the clinical trial registration number (at ClinicalTrials.gov or equivalent), where applicable.	NA
16. For phase II and III randomized controlled trials, please refer to the CONSORT flow diagram (see link list at top right) and submit the CONSORT checklist (see link list at top right) with your submission. See author guidelines, under 'Reporting Guidelines'. Please confirm you have submitted this list.	NA
17. For tumor marker prognostic studies, we recommend that you follow the REMARK reporting guidelines (see link list at top right). See author guidelines, under 'Reporting Guidelines'. Please confirm you have followed these guidelines.	NA

F- Data Accessibility

18: Provide a "Data Availability" section at the end of the Materials & Methods, listing the accession codes for data generated in this study and deposited in a public database (e.g. RNA-Seq data: Gene Expression Omnibus GSE39462, Proteomics data: PRIDE PXD000208 etc.) Please refer to our author guidelines for 'Data Deposition'.<br><br>Data deposition in a public repository is mandatory for:<br>a. Protein, DNA and RNA sequences<br>b. Macromolecular structures<br>c. Crystallographic data for small molecules<br>d. Functional genomics data<br>e. Proteomics and molecular interactions	RNA-seq data generated by this study have been deposited to GEO, accession # GSE157375.
19. Deposition is strongly recommended for any datasets that are central and integral to the study; please consider the journal's data policy. If no structured public repository exists for a given data type, we encourage the provision of datasets in the manuscript as a Supplementary Document (see author guidelines under 'Expanded View' or in unstructured repositories such as Dryad (see link list at top right) or Figshare (see link list at top right).	NA
20. Access to human clinical and genomic datasets should be provided with as few restrictions as possible while respecting ethical obligations to the patients and relevant medical and legal issues. If practically possible and compatible with the individual consent agreement used in the study, such data should be deposited in one of the major public access-controlled repositories such as dbGAP (see link list at top right) or EGA (see link list at top right).	NA
21. Computational models that are central and integral to a study should be shared without restrictions and provided in a machine-readable form. The relevant accession numbers or links should be provided. When possible, standardized format (SBML, CellML) should be used instead of scripts (e.g. MATLAB). Authors are strongly encouraged to follow the MIRIAM guidelines (see link list at top right) and deposit their model in a public database such as Biomodels (see link list at top right) or JWS Online (see link list at top right). If computer source code is provided with the paper, it should be deposited in a public repository or included in supplementary information.	NA

G- Dual use research of concern

22. Could your study fall under dual use research restrictions? Please check biosecurity documents (see link list at top right) and list of select agents and toxins (APHIS/CDC) (see link list at top right). According to our biosecurity guidelines, provide a statement only if it could.	No
