# Supplementary material for: SATB2‐LEMD2 interaction links nuclear shape plasticity to regulation of cognition‐related genes
Source: EMBO J. 2020 Dec 15;40(3):e103701. doi: 10.15252/embj.2019103701 (PMC7849313; doi:10.15252/embj.2019103701)
Supplement: Supplementary file 10 — Source Data for Figure 1 [file EMBJ-40-e103701-s009.zip › EMBOJ-2019-103701R_SourceDataFigure1_D.pdf]

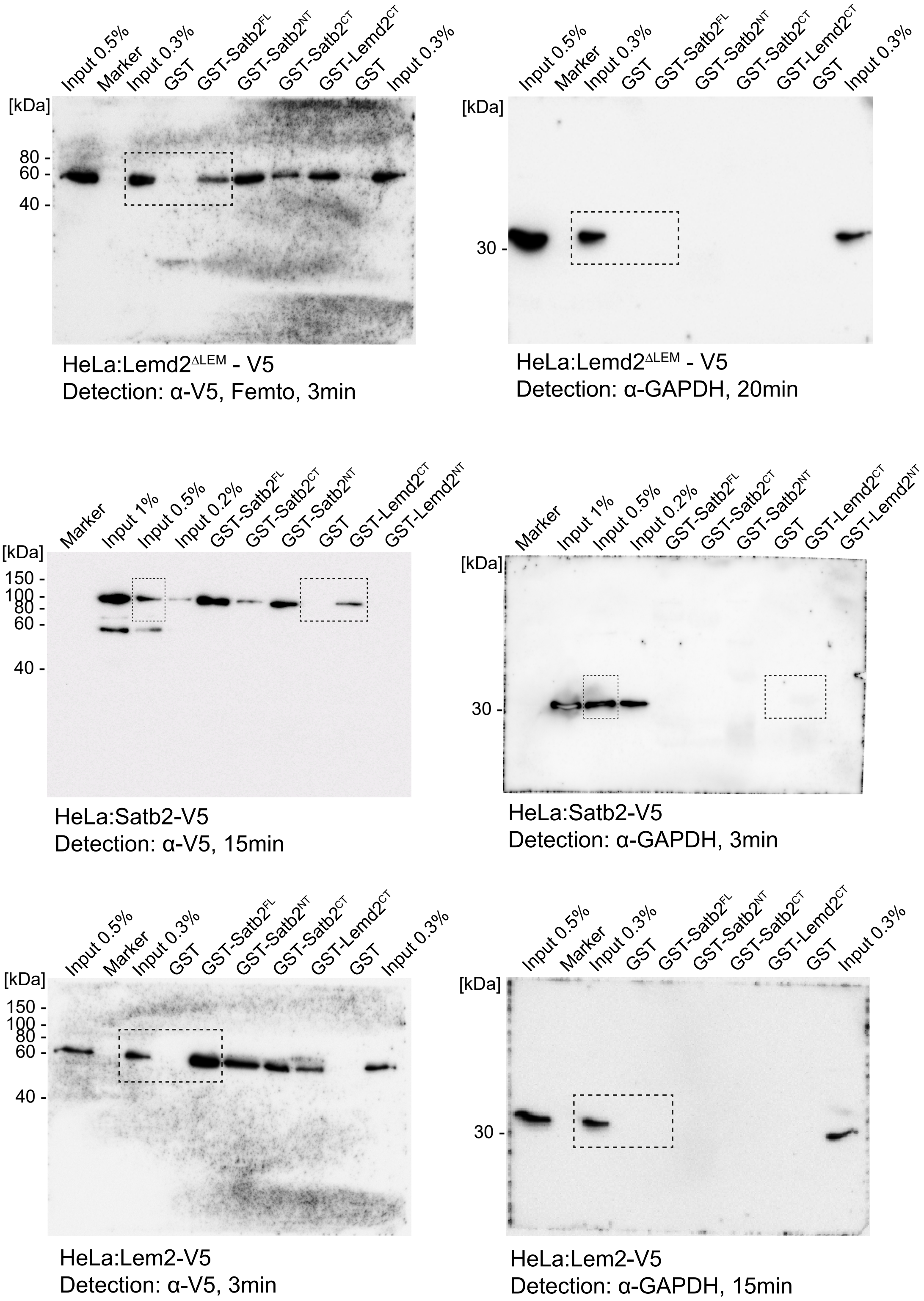

Source Data Figure 1 | Uncropped western blot membranes from pulldowns showing the protein:protein-interaction between SATB2 and LEMD2 (panel D).
